# Coat proteins of necroviruses target 14-3-3a to subvert MAPKKKα-mediated antiviral immunity in plants

**DOI:** 10.1038/s41467-022-28395-5

**Published:** 2022-02-07

**Authors:** Zongyu Gao, Dingliang Zhang, Xiaoling Wang, Xin Zhang, Zhiyan Wen, Qianshen Zhang, Dawei Li, Savithramma P. Dinesh-Kumar, Yongliang Zhang

**Affiliations:** 1 State Key Laboratory of Agro-Biotechnology and Ministry of Agriculture Key Laboratory of Soil Microbiology, College of Biological Sciences, China Agricultural University, 100193 Beijing, China; 2 Department of Plant Biology and The Genome Center, College of Biological Sciences, University of California, Davis, Davis, CA 95616 USA

**Keywords:** Virus-host interactions, Virulence, Plant signalling

## Abstract

Mitogen-activated protein kinase (MAPK) cascades play an important role in innate immunity against various pathogens in plants and animals. However, we know very little about the importance of MAPK cascades in plant defense against viral pathogens. Here, we used a positive-strand RNA necrovirus, beet black scorch virus (BBSV), as a model to investigate the relationship between MAPK signaling and virus infection. Our findings showed that BBSV infection activates MAPK signaling, whereas viral coat protein (CP) counteracts MAPKKKα-mediated antiviral defense. CP does not directly target MAPKKKα, instead it competitively interferes with the binding of 14-3-3a to MAPKKKα in a dose-dependent manner. This results in the instability of MAPKKKα and subversion of MAPKKKα-mediated antiviral defense. Considering the conservation of 14-3-3-binding sites in the CPs of diverse plant viruses, we provide evidence that 14-3-3-MAPKKKα defense signaling module is a target of viral effectors in the ongoing arms race of defense and viral counter-defense.

## Abstract (web summary)

MAPK cascades play an important role in innate immunity. Here, the authors show that that beet black scorch virus activates MAPK signaling in *Nicotiana benthamiana* and the viral coat protein counteracts MAPK-mediated defence by competitively binding to a MAPKKKα interactor.

## Introduction

In nature, plants are sessile stores of nutriment, thus frequently being attacked by numerous pests and pathogens, including insects, bacteria, fungi, and oomycetes. In order to survive, plants have developed a two-layered innate immune system to defend against invading pathogens^1^. Pattern-recognition receptors (PRRs) on the plasma membrane recognize pathogen/microbe-associated molecular patterns (PAMPs/MAMPs), triggering a series of downstream defense responses, including calcium flux, production of reactive oxygen species (ROS), activation of mitogen-activated protein kinase (MAPK) cascades and upregulation of defense genes. This perception of PAMPs/MAMPs constitutes the first layer of plant protection against pathogen attack and is referred to as pattern-triggered immunity (PTI)^2,3^. To establish successful infections, pathogens secrete effectors into the plant cell to suppress PTI, resulting in effector‐triggered susceptibility (ETS)^1^. Plants have evolved resistance (R) proteins to perceive these effectors and initiate a second layer of immunity named effector‐triggered immunity (ETI), which is generally stronger and lasting longer than PTI and often accompanied by hypersensitive responses (HR), a type of cell death to confine pathogens to the infection sites^4^.

It is widely accepted that RNA silencing is the first layer of plant defense against viruses^5,6^. In addition, plants also have evolved R proteins that recognize viral proteins as effectors and activate ETI similar to that employed in response to nonviral pathogens^7^. So far, no classical PAMPs have been found in plant–virus interactions. However, over the past decade, a growing body of evidence suggests that plants also deploy PTI to limit virus infection. For example, co-receptors such as brassinosteroid insensitive 1-associated kinase 1 (BAK1) and BAK1-like kinase 1 involved in PTI in *Arabidopsis thaliana* against nonviral pathogens are also shown to contribute to plant defense against several RNA viruses, including turnip crinkle virus (TCV)^8^, oilseed rape mosaic virus (ORMV), tobacco mosaic virus (TMV)^9^, and plum pox virus (PPV)^10^. MAPK4 homologs in soybean (*Glycine max*) are reported to negatively regulate SA accumulation and defense against soybean mosaic virus (SMV)^11^. Distinct from BAK1-mediated PTI, the nuclear shuttle protein-interacting kinase 1 (NIK1)-mediated antiviral signaling is specific to plant DNA viruses belonging to the genus *Begomovirus* of *Geminiviridae* family, and causes translocation of the ribosomal protein L10 (RPL10) to the nucleus for translational suppression of plant defense^12,13^. Geminivirus nuclear shuttle protein (NSP) overcomes this defense by interfering with NIK1-mediated nuclear relocalization of RPL10A^12^. Some RNA viruses have also been reported to counter PTI responses. For example, coat protein (CP) of PPV and movement protein (MP) of cucumber mosaic virus (CMV) were reported to suppress flg22-induced ROS production and PTI marker genes in *Arabidopsis* and *Nicotiana benthamiana*^10,14^. Cauliflower mosaic virus (CaMV) silencing suppressor P6 was shown to suppress ROS burst and SA-dependent autophagy^15^. Although these studies show some evidence suggesting an association of PTI in antiviral immunity, the functional role of PTI against plant viruses, especially positive-strand RNA viruses, and the molecular mechanisms underlying viral suppression of PTI remain to be fully characterized.

Although PTI and ETI are activated mainly through distinct immune receptors, they share some features of the signaling machinery and initiate many common responses^16,17^. For example, MAPK cascades serve as a convergent point downstream of PTI and ETI^18,19^. MAPK cascades are composed of three tiers of kinases, MAPKs–MAPKKs–MAPKKKs, and activated through phosphorylation by their upstream kinases in turn, which play a central role in stress signal transduction from upstream receptors to downstream targets^20^. Two main MAPK cascades are reported to be activated in *Arabidopsis* during PTI^21^, one is consisted of MAPKKK3/MAPKKK5-MKK4/MKK5-MPK3/MPK6^22,23^ and the other is consisted of MEKK1–MKK1/MKK2–MPK4^24,25^. In addition, several components of MAPK cascades are also reported to play important roles in R protein-mediated ETI and often associated with HR cell death. For example, in *Arabidopsis*, MPK3 and MPK6 are activated by RPS2-mediated immunity^26^. In tobacco, two MAPKs, salicylic acid-induced protein kinase (SIPK) and wounding-induced protein kinase (WIPK), the orthologs of MPK6 and MPK3 in *Arabidopsis*^20^, are involved in *N* gene-mediated resistance^27,28^ and cell death^29,30^. In tomato, two MAPKKs, MEK1 and MEK2, and two MAPKs, NTF6 and WIPK, contribute to Pto-mediated immunity^31^, and overexpression of a tomato MAPKKK, LeMAPKKKα, results in cell death^32^. Some pathogen effectors were reported to target MAPK cascades to inhibit defense responses such as AvrRpt2^33^, HopAI1^34^, and HopF2^35^. Recently, geminivirus tomato yellow leaf curl China virus (TYLCCNV) encoded βC1 was reported to directly target MKK2 and MPK4 to suppress plant immunity^36^. However, whether positive-strand RNA viruses target MAPK cascades remains to be determined.

In this study, we used a positive-strand RNA virus, beet black scorch virus (BBSV), as a model to explore the functional role of plant innate immune signaling in virus infection and the counter-defensive strategies employed by virus to overcome this layer of defense. We demonstrate that MAPKKKα-mediated defense against BBSV infection in *N. benthamiana* is correlated with the expression of a series of defense-related genes such as *PR1A*, *ERF1B*, and *HIR1*, whereas viral CP can subvert MAPKKKα-mediated antiviral innate immune response by targeting 14-3-3a. We also provide evidence that the CP targeting of MAPKKKα-14-3-3a defense signaling module also occurs in the infection of the other two plant RNA viruses in the family *Tombusviridae*. Our study provides mechanistic insight into understanding the suppression of plant innate immunity by RNA viruses.

## Results

### MAPKKKα plays an antiviral role during BBSV infection

**Fig. 1:**
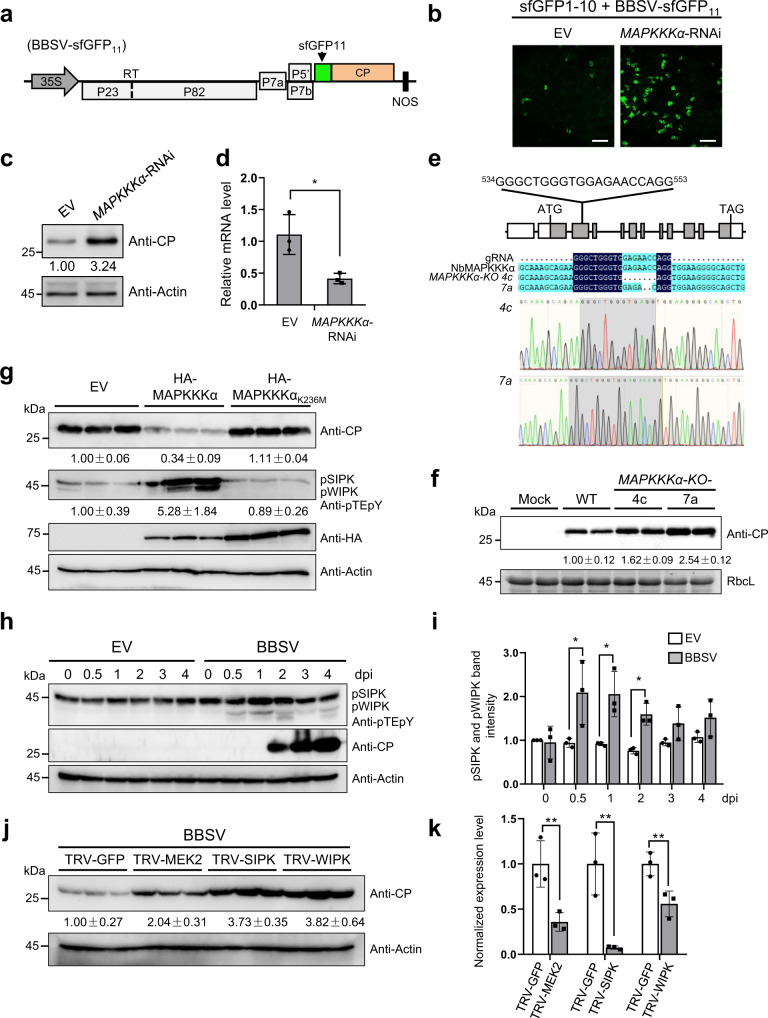
MAPKKKα plays an antiviral role during BBSV infection. **a** Schematic representation of BBSV-sfGFP_11_. 11th β-strand of super-folder GFP (sfGFP_11_) is fused to the N-terminus of CP in BBSV infectious clone. **b** Knockdown of MAPKKKα promotes BBSV infection. *Agrobacterium* containing hairpin MAPKKKα or the control empty vector (EV) was infiltrated into half of *N. benthamiana* leaves, respectively. Twenty-four hours later, a mixture of *Agrobacterium* harboring BBSV-sfGFP_11_ and sfGFP1-10 constructs was infiltrated into the pre-infiltrated leaves. GFP fluorescence was visualized by confocal microscopy at 5 dpi. Scale bars = 100 µm. **c** The expression level of BBSV CP in the infiltrated leaves was examined by western blot analysis using an anti-CP antibody. Actin served as the loading control. For panels **b** and **c**, the experiments were repeated three times with similar results. **d** RT-qPCR to confirm the downregulation of MAPKKKα. Values represent  ± SD of the mean from three biological replicates. An asterisk indicates the significant difference (*P = 0.0104) based on one-sided unpaired Student’s *t* test. **e** Locus of the single guide RNA target in the genomic fragment of NbMAPKKKα. Exons are represented by gray boxes, UTRs by white boxes, and introns by black lines. In T2 transformants, deletions were detected in two *NbMAPKKKα*-KO lines. **f** Western blot analysis of BBSV CP levels in the infiltrated leaves of wild-type (WT) or *NbMAPKKKα*-KO plants using an anti-CP antibody. RbcL served as the loading control. The experiment was repeated three times with similar results. **g** Overexpression of MAPKKKα inhibits BBSV infection. *Agrobacterium* containing MAPKKKα or MAPKKKα_K236M_ or the control empty vector (EV) was infiltrated into different *N. benthamiana* leaves. *Agrobacterium* harboring BBSV infectious clone was infiltrated 24 h later. The accumulation levels of BBSV or phosphorylated NbSIPK and NbWIPK were detected by western blot analysis with an anti-CP or anti-phospho-p44/42 MAPKs (anti-pTEpY) antibody. Actin protein served as the loading control. Each lane represents a sample from individual experiments. h. MAPK activation in BBSV-infected *N. benthamiana*. Eight-leaf-stage *N. benthamiana* plants were infiltrated with *Agrobacterium* carrying empty vector (EV) or BBSV infectious clone. Infiltrated leaves were collected at indicated time points and subjected to western blot analysis with an anti-pTEpY or anti-BBSV CP antibody. Actin protein served as the loading control. **i** Quantification of bands from blot in panel **h**, with pSIPK and pWIPK abundance normalized to the intensity of EV control at 0 h. Values represent ± SD of the mean from three biological replicates. An asterisk indicates the significant difference (*P = 0.0498, 0.0284, 0.0134, respectively) based on one-sided paired Student’s *t* test. **j** Western blot analysis of BBSV CP accumulation in the TRV-*GFP* control, TRV-*NbMEK2*, TRV-*NbSIPK*, and TRV-*NbWIPK* inoculated *N. benthamiana* plants. Actin protein served as the loading control. **k** RT-qPCR to confirm the downregulation of *NbMEK2*, *NbSIPK*, and *NbWIPK*. Values represent ± SD of the mean from three biological replicates. An asterisk indicates the significant difference (**P = 0.0043, 0.0068, 0.0085, respectively) based on one-sided unpaired Student’s *t* test. For panels **h** and **j**, the experiments were repeated three times with similar results.

MAPKKKα has been reported to positively regulate cell-death signaling associated with plant immunity^32^. However, little is known about its role in virus infection. We used BBSV as a model to explore the role of MAPKKKα in plant–virus infection. To monitor the virus infection, we used a self-assembling split super-folder green fluorescent protein (sfGFP) system^37^. In this system, 11th β-strand of sfGFP (sfGFP11) was fused to the N-terminus of CP in the BBSV infectious clone to generate BBSV-sfGFP_11_ (Fig. 1a). Co-expression of BBSV-sfGFP_11_ with sfGFP1-10 β-strand (sfGFP1-10) in plant cells will result in the GFP fluorescence, which can be used to indicate the infection of BBSV. We then knocked down the expression of MAPKKKα by using intron spliced hairpin RNAi vector-mediated gene silencing^38^, and observed increased GFP fluorescence and CP accumulation in *MAPKKKα*-RNAi leaves compared with the empty vector (EV) control (Fig. 1b, c). Downregulation of MAPKKKα using this strategy was validated by RT-qPCR (Fig. 1d). To further confirm these results, we generated *N. benthamiana MAPKKKα*-knockout (KO) plants via CRISPR-Cas9 gene editing. MAPKKKα has a homolog (MAPKKKα-2) in *N. benthamiana* (Supplementary Fig. 1a) and Sanger sequencing indicated deletions at gRNA targeting site within two *MAPKKKα* genes in *MAPKKKα-*KO plants (Fig. 1e and Supplementary Fig. 1b). *MAPKKKα*-KO plants accumulated increased CP upon BBSV inoculation (Fig. 1f). These results indicate that MAPKKKα plays an antiviral role during BBSV infection.

To further determine the role of MAPKKKα in BBSV infection, we overexpressed MAPKKKα and a kinase-inactive mutant, MAPKKKα_K236M_ (K236 is a conserved ATP binding site essential for the kinase activity of MAPKKKα^32^) followed by BBSV inoculation. At 3-day post infiltration (dpi), the accumulation of BBSV CP was decreased in leaves overexpressing MAPKKKα compared with EV control. However, overexpression of MAPKKKα_K236M_ had no significant effect on BBSV CP accumulation (Fig. 1g, top panel). Moreover, the phosphorylation levels of two MAPKs, NbSIPK, and NbWIPK, which were reported to act downstream of MAPKKKα^32^ were higher in the leaves overexpressing MAPKKKα compared with EV and MAPKKKα_K236M_ (Fig. 1g, middle panel). Furthermore, the expression of MAPKKKα induced cell death but not by MAPKKKα_K236M_ (Supplementary Fig. 2). The expression of MAPKKKα and MAPKKKα_K236M_ was confirmed by western blot (Fig. 1g, middle panel). These results suggest that MAPKKKα may negatively regulate BBSV infection via activation of the downstream MAPK cascades.

To analyze whether MAPK cascades were activated during BBSV infection, we infiltrated *N. benthamiana* with *Agrobacterium* containing BBSV infectious cDNA and monitored the phosphorylation status of NbSIPK and NbWIPK at different time points. We observed a significant increase in phosphorylation levels of NbSIPK and NbWIPK at 0.5-, 1-, and 2-day post BBSV inoculation (Fig. 1h, i). These results indicate that the MAPK cascade is activated at the early stage of BBSV infection.

To further test whether the activated MAPK signaling is involved in defense against BBSV infection, we performed loss-of-function assays of *NbMEK2*, *NbSIPK*, and *NbWIPK* by using *Tobacco rattle virus* (TRV)-based gene silencing (VIGS). CP accumulation in *NbMEK2*, *NbSIPK*, or *NbWIPK* silenced plants was higher than in the control TRV-GFP inoculated plants (Fig. 1j). The silencing efficiency of *NbMEK2*, *NbSIPK*, or *NbWIPK* was confirmed by RT-qPCR (Fig. 1k). Together, these results indicate that BBSV infection activates the MAPKKKα-MEK2-SIPK/WIPK cascade, which in turn induces plant defense responses against BBSV infection.

### MAPKKKα-mediated antiviral response associates with the expression of defense-related genes

**Fig. 2:**
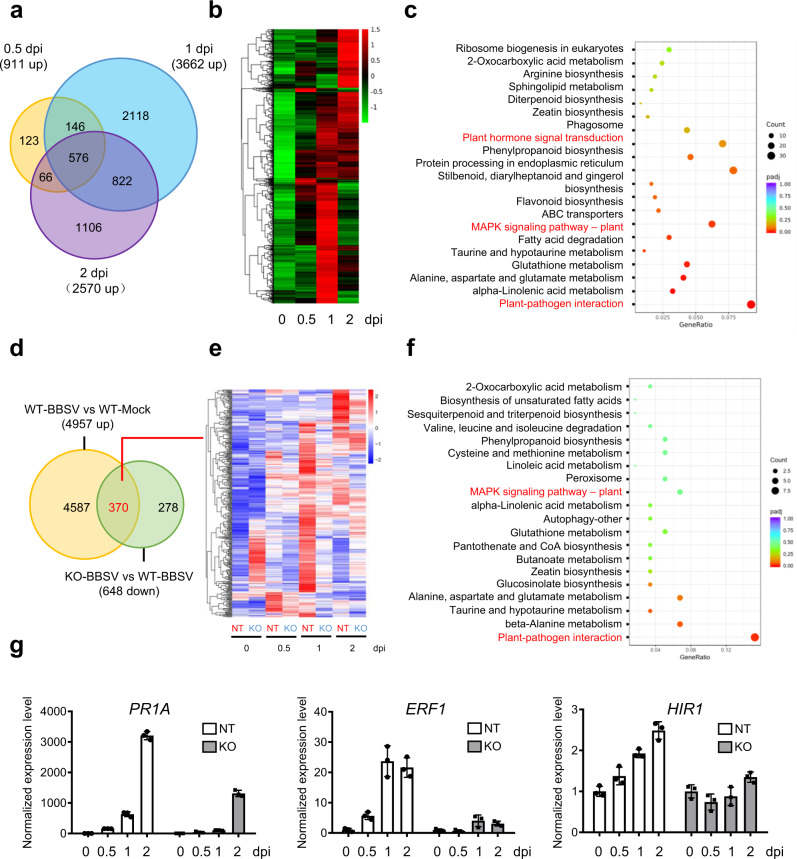
RNA-seq analyses of non-transgenic and MAPKKKα-KO plants in response to BBSV infection. **a** Venn diagrams showing overlaps of upregulated genes in WT-BBSV at different time points vs WT-Mock. **b** Heatmaps showing hierarchical cluster analysis of all differentially expressed genes. Data were collected from three biological replicates, each containing a pool of three individual plants. The resulting *P* values were adjusted using Benjamini and Hochberg’s approach for controlling the false discovery rate (padj). *P*adj < = 0.05 and |log2(foldchange)| > = 1 were set as the threshold for significantly differential expression. **c** The Kyoto Encyclopedia of Genes and Genomes (KEGG) enrichment analyses of all upregulated genes in BBSV-infected plants. **d** Venn diagrams showing overlaps of 4957 upregulated genes in WT-BBSV vs WT-Mock and 648 downregulated genes in KO-BBSV vs WT-BBSV. **e** Heatmaps showing hierarchical cluster analysis of 370 differentially expressed genes shown in (**d**). **f** KEGG enrichment analyses of 370 differentially expressed genes shown in (**d**). **g** RT-qPCR assay to validate the accuracy of the transcriptome data for these differentially expressed genes shown in (**d**). *EF-1α* was used as the internal reference gene to normalize the relative expression. Values are mean ± SD from three biological repeats.

To further investigate the mechanism underlying MAPKKKα-mediated antiviral response, we performed RNA-seq analysis in wild-type (WT) and the *MAPKKKα*-KO plants during BBSV infection. We collected leaves from mock- and BBSV-infected plants at 0.5, 1, and 2 dpi and performed RNA-Seq. We observed upregulation of 911, 3662, and 2570 genes in WT plants at 0.5, 1, and 2 dpi, respectively (Fig. 2a, b and Supplementary Data 1). The Kyoto Encyclopedia of Genes and Genomes (KEGG) enrichment analysis indicated that these upregulated genes were highly enriched in plant–pathogen interaction process, MAPK signaling pathway, and plant hormone signal transduction (Fig. 2c). Further analysis showed that 370 genes, which were upregulated in WT plants after BBSV infection, were downregulated in the comparisons KO-BBSV vs WT-BBSV (Fig. 2d, e and Supplementary data 2). KEGG analysis revealed that these downregulated genes were also enriched in plant–pathogen interaction process and MAPK signaling pathway (Fig. 2f). We selected three defense-related genes, including *PATHOGENESIS-RELATED PROTEIN 1A* (*PR1A*), *ETHYLENE-RESPONSIVE TRANSCRIPTION FACTOR 1B* (*ERF1B*)^39^, and *HYPERSENSITIVE-INDUCED RESPONSE PROTEIN 1* (*HIR1*)^40^ for further RT-qPCR analysis, and the results were consistent with the RNA-seq data (Fig. 2g). Together, these results suggest that MAPKKKα-mediated defense against BBSV infection is correlated with the expression of defense-related genes.

### BBSV CP suppresses MAPKKKα-mediated antiviral immunity through its interaction with 14-3-3a

**Fig. 3:**
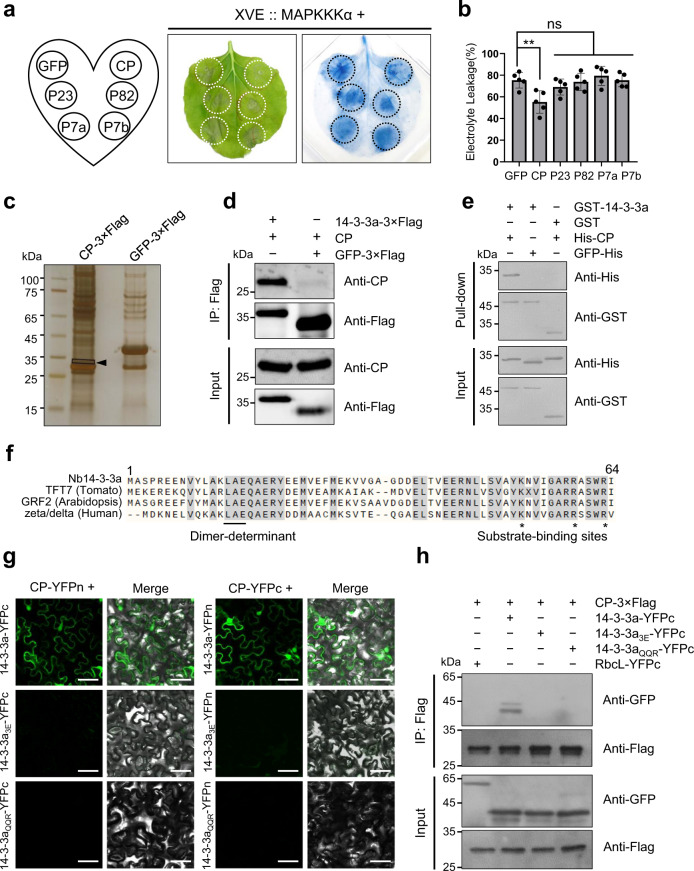
CP suppresses MAPKKKα-induced cell death by interacting with 14-3-3a. **a** BBSV CP suppresses the cell death induced by MAPKKKα. Five different BBSV-encoded proteins or GFP control were co-expressed with HA-MAPKKKα controlled by an estradiol-inducible system in indicated leaf regions. β-estradiol was infiltrated into the leaves at 48 h after agroinfiltration. Leaves were stained by trypan blue, and representative photographs were taken at three days after estradiol treatment. **b** Quantification of cell death by measuring electrolyte leakage of leaves shown in (**a**). Error bars indicate ± SD of the mean (*n* = 5 biologically independent plants). Asterisks indicated the significant difference based on one-way ANOVA analysis with Dunnett’s multiple comparison test (**P = 0.0013). **c** Silver-stained SDS-PAGE gel image of CP-3×Flag and GFP-3×Flag protein immunoprecipitated using anti-Flag beads from BBSV-infected *N. benthamiana* leaf tissues. The band indicated by the rectangle was analyzed by LC-MS/MS. **d** Co-IP analysis of the interaction between 14-3-3a and CP. *N. benthamiana* leaves transiently expressing combinations of different proteins indicated above the panel were harvested at 3 dpi. Total proteins were immunoprecipitated with anti-Flag beads and detected by western blot with an anti-CP or anti-Flag antibody. **e** GST pull-down assay to detect the interaction between CP and 14-3-3a. Purified His-CP or His-GFP was incubated with GST-14-3-3a or GST protein control. After incubation with glutathione-Sepharose beads, the pull-down products were analyzed by western blot with an anti-His or anti-GST antibody. For panels **c**–**e**, the experiments were repeated three times with similar results. **f** Depiction of 14-3-3 N-terminal sequences alignment generated by UniPort (https://www.uniprot.org/align/). 14-3-3 isoforms from different species were indicated on the left side. The underlined amino acids are required for dimerization of 14-3-3 proteins^44^. The amino acids responsible for 14-3-3-ligand-binding activity are highlighted with asterisks^43^. **g** BiFC analysis of the interaction between wild-type or mutant 14-3-3a and CP. CP and wild-type or mutant 14-3-3a fused to N or C-terminus of YFP were transiently co-expressed in *N. benthamiana* leaves. Confocal analysis was performed at 3 dpi. Representative results of at least three independent experiments are shown. Scale bars = 50 µm. **h** Co-IP analysis of the interaction between wild-type or mutant 14-3-3a and CP. *N. benthamiana* leaves transiently expressing combinations of different proteins were harvested at 3 dpi. Total proteins were immunoprecipitated with anti-Flag beads and detected by western blot with an anti-GFP or anti-Flag antibody. The experiment was repeated three times with similar results.

We noted that the phosphorylation levels of NbSIPK and NbWIPK declined two days after BBSV infection (Fig. 1h, i), suggesting that BBSV may have evolved with strategies to suppress MAPK activity. To test if BBSV-encoded proteins could suppress MAPKKKα-mediated signal transduction, we co-expressed different BBSV proteins and GFP control with HA-MAPKKKα, which was controlled by an estradiol-inducible system. Trypan blue staining of leaf tissues and the electrolyte-leakage assay showed that only CP of BBSV suppressed MAPKKKα-induced cell death while other viral proteins showed a similar cell-death phenotype to that of the GFP control (Fig. 3a, b). Western blot analysis with anti-GFP antibody confirmed the expression of these proteins (Supplementary Fig. 3a). Together, these results demonstrate that BBSV CP plays a role in suppressing MAPKKKα-mediated defense.

We reasoned that CP may suppress MAPKKKα-mediated defense by directly interacting with MAPKKKα and inhibiting its activity. To test this, we performed bimolecular fluorescence complementation (BiFC) assay. As a positive control, CP-YFPn and CP-YFPc resulted in strong reconstituted YFP signals, yet no fluorescence signal was observed in the leaves co-expressing either CP-YFPc and MAPKKKα_K236M_-YFPn or CP-YFPn and MAPKKKα_K236M_-YFPc (Supplementary Fig. 4a). The expression of proteins was confirmed by western blot analysis (Supplementary Fig. 3b, c). Yeast two-hybrid (Y2H) assay showed that yeast expressing AD-MAPKKKα and BD-CP or AD-CP and BD-MAPKKKα failed to grow on the drop-out media compared with the positive control (Supplementary Fig. 4b). These results indicate that CP does not directly interact with MAPKKKα.

To identify the targets of BBSV CP that could interfere with the MAPKKKα activity, we performed immunoprecipitation (IP) of CP-3×FLAG and separated immunoprecipitates by SDS-PAGE followed by silver staining. A distinct band was visible in the IP products from CP-3×FLAG but the corresponding band was absent in the lane of GFP-3×FLAG control (Fig. 3c). The band was excised from the gel and analyzed by liquid chromatography coupled to tandem mass spectrometry (LC-MS/MS). Several 14-3-3 proteins were identified in the MS dataset (Supplementary Data 3). Considering the important role of 14-3-3 proteins in plant immunity^41,42^, we hypothesized that 14-3-3 proteins might take part in the plant response to BBSV infection. Among identified 14-3-3 proteins, *N. tomentosiformis* 14-3-3-like protein A (gi|697111701) showed the highest score, with 100% amino acid identity with *N. benthamiana* 14-3-3a (Supplementary Fig. 5). We then cloned 14-3-3a from *N. benthamiana* and used it as a surrogate for further analysis.

To investigate whether CP interacts with 14-3-3a in planta, we cloned full-length 14-3-3a from *N. benthamiana* and performed co-immunoprecipitation (Co-IP) assay. We found that CP coimmunoprecipitates with 14-3-3a-3×FLAG, but not with GFP-3×FLAG control (Fig. 3d). BiFC experiments confirmed the interaction in planta (Fig. 3g, top panel). To see if CP interacts directly with 14-3-3a, we performed a GST pull-down assay. A GST tag was fused to the N-terminus of 14-3-3a and a 6xHis tag was fused to CP. GST and GFP-His served as negative controls. The result showed that GST-14-3-3a specifically binds to the His-CP but not to the GFP-His control (Fig. 3e). Based on these results, we conclude that CP interacts with 14-3-3a in vivo and in vitro.

There are several conserved amino acids within animal 14-3-3s protein that determine its binding to the substrate^43^ and dimerization^44^. Amino acid sequence alignment indicates that the amino acids required for these functions are also conserved in plant 14-3-3s (Fig. 3f). To examine whether these amino acids are responsible for 14-3-3 dimerization and its binding to the CP, two mutants, 14-3-3a_QQR_, in which the amino acids at 12-LAE-14 was mutated to QQR, and 14-3-3a_3E_, in which the amino acids of K52, R59, and R63 were all mutated to E, were constructed. Only the mutation in LAE eliminated the dimerization of 14-3-3a (Supplementary Fig. 6), and both of these two mutants fail to interact with CP as shown in the BiFC and Co-IP assays (Fig. 3g, h). The expression of proteins in BiFC assay was confirmed by western blot analysis (Supplementary Fig. 3d). These results indicate that both the dimerization and substrate binding activity of 14-3-3a is indispensable for its binding to CP.

### Nb14-3-3a positively regulates MAPKKKα-mediated cell death

**Fig. 4:**
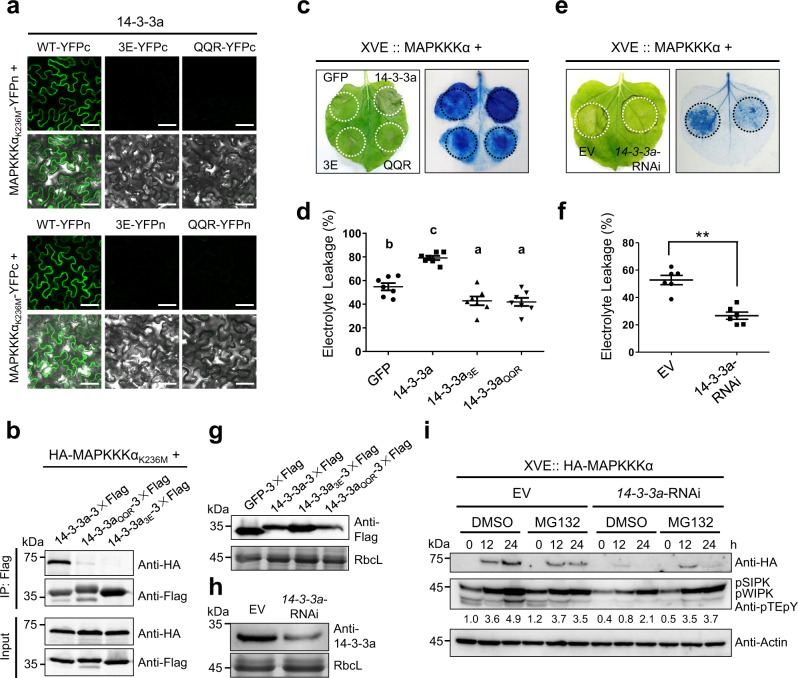
14-3-3a positively regulates MAPKKKα-induced cell death. **a** BiFC analysis of the interaction between wild-type or mutant 14-3-3a and MAPKKKα. Combinations of different proteins were transiently expressed in *N. benthamiana* leaves. Confocal analysis was performed at 3 dpi. Representative results of at least three independent experiments are shown. Scale bars = 50 µm. **b** Co-IP analysis of the interaction between wild-type or mutant 14-3-3a and MAPKKKα_K236M_. *N. benthamiana* leaves transiently expressing combination of different proteins were harvested at 3 dpi. Total proteins were immunoprecipitated with anti-Flag beads and detected by western blot with an anti-HA or anti-Flag antibody. The experiment was repeated three times with similar results. **c** Overexpression of 14-3-3a enhances MAPKKKα-induced cell death. Indicated proteins together with HA-MAPKKKα controlled by an estradiol-inducible system were co-expressed in different leaf regions. β-estradiol was applied to the leaves 48 h after agroinfiltration. Leaves were stained by trypan blue and representative photographs were taken 3 days after estradiol treatment. **d** Quantification of cell death by measuring electrolyte leakage of leaf regions shown in panel (**c**). Error bars indicate ± SD of the mean (*n* = 7 biologically independent plants). Different letters in the chart indicate statistically significant differences among different groups according to the one-way ANOVA analysis with Tukey’s multiple comparison test (*P* < 0.05). **e** Knockdown of 14-3-3a eliminates MAPKKKα-induced cell death. *Agrobacterium* containing hairpin 14-3-3a or the control empty vector was co-infiltrated with HA-MAPKKKα controlled by an estradiol-inducible system into the opposite halves of *N. benthamiana* leaves. β-estradiol was applied to the leaves 48 h after agroinfiltration. Leaves were stained by trypan blue and representative photographs were taken 3 days after estradiol treatment. **f** Quantification of cell death by measuring electrolyte leakage of leaves treated as described in (**e**). Error bars indicate ± SD of the mean (*n* = 6 biologically independent plants). Asterisks indicate the significant difference (**P = 0.0024) based on one-sided paired Student’s *t* test. **g** Western blot analysis to confirm the expression of the GFP and 14-3-3a proteins shown in panel **c** with an anti-Flag antibody. **h** Western blot analysis to confirm the downregulation of 14-3-3a at protein level shown in panel **e** with an anti-14-3-3a antibody. **i** 14-3-3a is important for maintaining the stability of MAPKKKα. *Agrobacterium* containing hairpin 14-3-3a or empty vector were co-infiltrated with HA-MAPKKKα controlled by an estradiol-inducible system into the leaves. In total, 30 μM β-estradiol with 50 μM MG132 or DMSO (control) was infiltrated into the leaves 48 h after agroinfiltration. Samples were collected at 0, 12, 24 h after treatment with a chemical reagent, and subjected to western blot with an anti-HA or anti-pTEpY antibody. For panels **g**–**i**, the experiments were repeated three times with similar results.

To test whether 14-3-3a plays a functional role in the activation of cell death through MAPKKKα in *N. benthamiana*, we performed BiFC and Co-IP assays. The results showed that the wild-type 14-3-3a, but not the 14-3-3a_3E_ and 14-3-3a_QQR_ mutants, interacts with MAPKKKα (Fig. 4a, b). The expression of proteins in BiFC assay was confirmed by western blot analysis (Supplementary Fig. 3b, c). We then examined the effect of 14-3-3a on MAPKKKα-induced cell death. Wild-type or mutant 14-3-3a was co-expressed with estradiol-induced HA-MAPKKKα protein, and cell death was evaluated at 3 dpi after 12 h estradiol treatment. Trypan blue staining showed that overexpression of wild-type 14-3-3a significantly enhanced MAPKKKα-induced cell death compared with the GFP control (Fig. 4c). In contrast, co-expression of either 14-3-3a_3E_ or 14-3-3a_QQR_ mutant with HA-MAPKKKα showed an alleviated cell-death phenotype (Fig. 4c). An electrolyte-leakage assay substantiated the results shown in Fig. 4c by showing that electrolyte leakage was increased by about 25% with the expression of wild-type 14-3-3a and decreased by about 10% with the expression of 14-3-3a mutants compared with the GFP control (Fig. 4d), indicating a possible dominant-negative effect of these two mutants on MAPKKKα-induced cell death. The expression of wild-type 14-3-3a, mutant, and GFP proteins was confirmed by western blot analysis (Fig. 4g). We next tested the MAPKKKα-induced cell death in the *Nb14-3-3a*-silenced leaves. Trypan blue staining and the electrolyte-leakage assay showed that MAPKKKα-induced cell death was dramatically decreased when *14-3-3a* was silenced (Fig. 4e, f). Downregulation of 14-3-3a by RNAi was confirmed by western blot analysis with anti-14-3-3a antibody (Fig. 4h). These results indicate that 14-3-3a functions as a positive regulator of MAPKKKα-induced cell death.

We also found that the downregulation of *14-3-3a* affects the accumulation of MAPKKKα protein, which can be partially rescued by the addition of the proteasome inhibitor MG132 (Fig. 4i). SIPK and WIPK phosphorylation levels were in line with MAPKKKα protein levels (Fig. 4i). RT-qPCR analysis showed that silencing of *Nb14-3-3a* had no obvious effects on the transcription of *NbMAPKKKα* (Supplementary Fig. 7), suggesting that Nb14-3-3a mainly regulated the abundance of NbMAPKKKα at the protein level. Together, these results demonstrate that 14-3-3a enhances MAPKKKα-mediated signaling by increasing the stability of MAPKKKα.

### Nb14-3-3a-mediated antiviral defense depends on the MAPKKKα

**Fig. 5:**
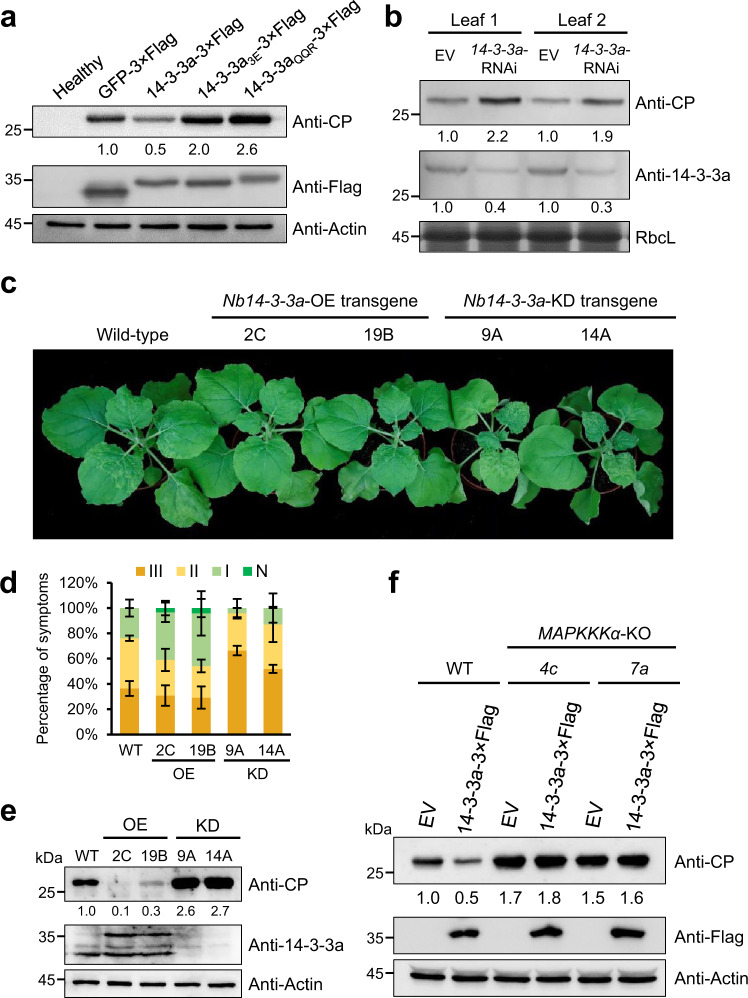
Nb14-3-3a-mediated antiviral defense depends on the MAPKKKα. **a** Overexpression of 14-3-3a inhibits BBSV infection. *Agrobacterium* containing indicated construct above the panel was infiltrated into *N. benthamiana* leaves followed by mechanical inoculation with 300 ng BBSV virions 24 h later. Inoculated leaves were collected at 3 dpi and analyzed by western blot analysis with an anti-CP or anti-Flag antibody. Actin served as the loading control. **b** Knockdown of *Nb14-3-3a* increases the accumulation of BBSV. The opposite halves of *N. benthamiana* leaves were agroinfiltrated with *14-3-3a*-RNAi construct and the control empty vector (EV). *Agrobacterium* carrying BBSV infection clone was infiltrated into the previously infiltrated regions 24 h later. Leaf samples from infiltrated regions were harvested at 3 dpi and subjected to western blot with an anti-CP or anti-14-3-3a antibody. CBB-stained RbcL served as the loading control. For panels **a** and **b**, the experiments were repeated three times with similar results. **c** Phenotypes of BBSV-infected WT, *14-3-3a*-OE, and *14-3-3a*-KD plants. *N. benthamiana* leaves were inoculated with 100 ng BBSV virions. Photographs were taken at 14 dpi and a representative result is shown. **d** The percentages of BBSV-infected wild-type (WT), *14-3-3a*-OE, and *14-3-3a*-KD plants with different disease symptom grades. Error bars indicate ± SD of the mean three independent experiments (*n* = 3) with at least six plants in each experiment. **e** Western blot analysis of BBSV CP levels in the indicated plants using an anti-CP or anti-14-3-3a antibody. Actin served as the loading control. **f** Western blot analysis of BBSV CP levels in the infiltrated leaves of WT or *NbMAPKKKα*-KO plants using an anti-CP or anti-Flag antibody. Actin served as the loading control. For panels **e** and **f**, the experiments were repeated three times with similar results.

To determine the functional role of 14-3-3a in viral infection, we transiently overexpressed wild-type and mutant 14-3-3a in *N. benthamiana* leaves and then mechanically inoculated BBSV virions at 24 h post infiltration (hpi). The accumulation of BBSV CP was greatly reduced in the leaves overexpressing wild-type 14-3-3a compared with the control leaves expressing GFP (Fig. 5a), indicating that overexpression of 14-3-3a inhibits BBSV infection. In contrast, we found increased CP accumulation in leaves overexpressing 14-3-3a mutants that fail to interact with CP (Fig. 5a), suggesting a dominant-negative effect of these mutants on BBSV infection. We also transiently silenced 14-3-3a followed by infiltration with *Agrobacterium* harboring the BBSV infectious clone at 24 hpi. Western blotting with anti-CP or anti-14-3-3a antibodies showed that the accumulation of viral CPs was increased along with the downregulation of 14-3-3a expression (Fig. 5b). To further confirm the role of 14-3-3a in BBSV infection, we generated 14-3-3a overexpression (*14-3-3a*-OE) and knockdown (*14-3-3a*-KD) transgenic *N. benthamiana* plants. We then compared the symptoms of WT and transgenic *N. benthamiana* plants upon BBSV infection. The symptoms of BBSV-infected plants were arbitrarily divided into four grades based on the severity of symptoms on systemic leaves (Supplementary Fig. 8). When these transgenic plants were inoculated with BBSV virions, we found that a higher percentage of *14-3-3a*-OE plants showed attenuated symptoms (grade I) and a lower percentage of plants showed severe disease symptoms (grade III) in the systemic leaves. In contrast, BBSV induced more severe symptoms in *14-3-3a*-KD plants compared with that of the control WT plants (Fig. 5c, d). Consistently, BBSV CP accumulated to a significantly lower level in *14-3-3a*-OE plants, whereas CP accumulation was increased more than twice in *14-3-3a*-KD plants in comparison with the non-transgenic plants (Fig. 5e). Together, these results demonstrate that 14-3-3a plays an antiviral role in BBSV infection.

Based on the data presented above, we reasoned that 14-3-3a-mediated defense against BBSV might depend on MAPKKKα. To test this hypothesis, we overexpressed 14-3-3a in WT and *MAPKKKα-*KO plants, and performed BBSV infection assay. Western blot analysis showed that overexpression of 14-3-3a in *MAPKKKα-*KO plants failed to impair the BBSV infection based on the CP accumulation, in contrast to that of the WT plants (Fig. 5f). These results suggest that 14-3-3a positively regulates antiviral defense in a MAPKKKα-dependent manner.

### CP suppresses the MAPKKKα-mediated cell-death signaling by targeting Nb14-3-3a

**Fig. 6:**
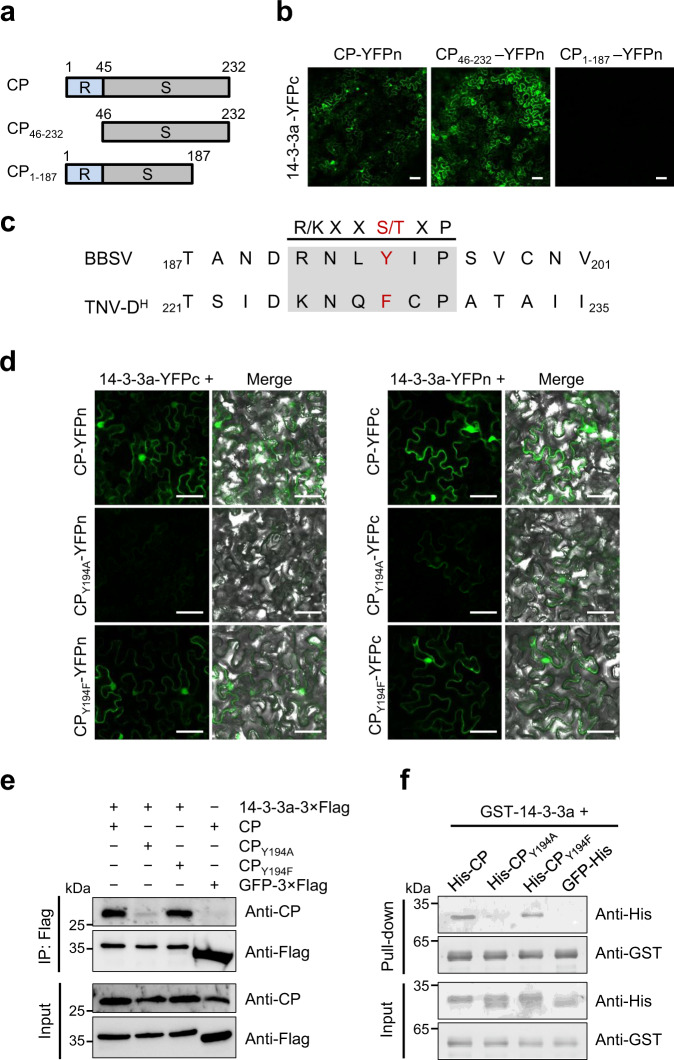
Determination of key amino acids that are responsible for CP-14-3-3a interaction. **a** Schematic representation of CP truncated mutants used for BiFC assays. **b** BiFC analysis of regions within CP that interacted with 14-3-3a. Wild-type or truncated CP mutants fused to N-terminus of YFP and 14-3-3a fused to C-terminus were transiently co-expressed in *N. benthamiana* leaves. Confocal analysis was performed at 3 dpi. Representative results of at least three independent experiments are shown. Scale bars = 50 µm. **c** Multiple sequence alignment of the C-terminus of CPs from Betanecroviruses. The 14-3-3-binding-like motif is shaded in gray, and the putative 14-3-3-binding site is highlighted in red. **d** BiFC analysis of the interaction between wild-type or mutant CP and 14-3-3a. Wild-type or mutant CP and 14-3-3a fused to N or C-terminus of YFP were transiently co-expressed in *N. benthamiana* leaves. Confocal analysis was performed at 3 dpi. Representative results of at least three independent experiments are shown. Scale bars = 50 µm. **e** Co-IP analysis of the interaction between wild-type or mutant CP and 14-3-3a. *N. benthamiana* leaves transiently expressing combinations of different proteins were harvested at 3 dpi. Total proteins were immunoprecipitated with anti-Flag beads and detected by western blot with an anti-CP or anti-Flag antibody. **f** GST pull-down assay to detect the interaction between wild-type or mutant CP and 14-3-3a. Purified His-tagged CP or its derivatives was incubated with GST-14-3-3a or GST protein. His-GFP protein served as the negative control. After incubation with glutathione-Sepharose beads, the pull-down products were analyzed by western blot with an anti-His or anti-GST antibody. For panels **e** and **f**, the experiments were repeated three times with similar results.

BBSV CP is divided into two regions: a N-terminal region responsible for RNA binding and nuclear localization^45,46^, and a shell domain^47^. To determine the region of CP protein that is important for its binding to 14-3-3a, we constructed N-terminal or C-terminal truncated CP mutants respectively (Fig. 6a), and tested their interactions with 14-3-3a by BiFC assay. The result showed that CP_46-232_ retains the ability to interact with 14-3-3a, whereas CP_1-187_ was unable to interact with 14-3-3a (Fig. 6b). The expression of proteins was confirmed by western blot analysis (Supplementary Fig. 3e). These results indicate that the C-terminal region between amino acids 188 and 232 is required for CP interaction with 14-3-3a. By aligning CP sequences of BBSV and TNV-D^H^, we found a motif in this region, which is similar to a conventional 14-3-3-binding motif (Rxx[pS/pT]xP)^48^. Intriguingly, this motif in BBSV CP contains tyrosine (Y) instead of S/T and TNV-D^H^ contains phenylalanine (F) (Fig. 6c). The Y and F amino acids are structurally similar, implying that the phosphorylation of the 14-3-3-binding motif in BBSV CP may not be required for its binding to 14-3-3a and the maintenance of the structure may be critical for its binding. To test this hypothesis, we mutated Y194 in BBSV CP into F and alanine (A) and tested their binding abilities to 14-3-3a. BiFC, Co-IP, and GST pull-down assays showed that CP_Y194F_ mutant retains the ability to interact with 14-3-3a, whereas CP_Y194A_ mutant did not (Fig. 6d–f). The expression of proteins in BiFC assay was confirmed by western blot analysis (Supplementary Fig. 3e). Together, these data indicate that the conformation but not the phosphorylation state of Y194 of CP is important for its interaction with 14-3-3a.

**Fig. 7:**
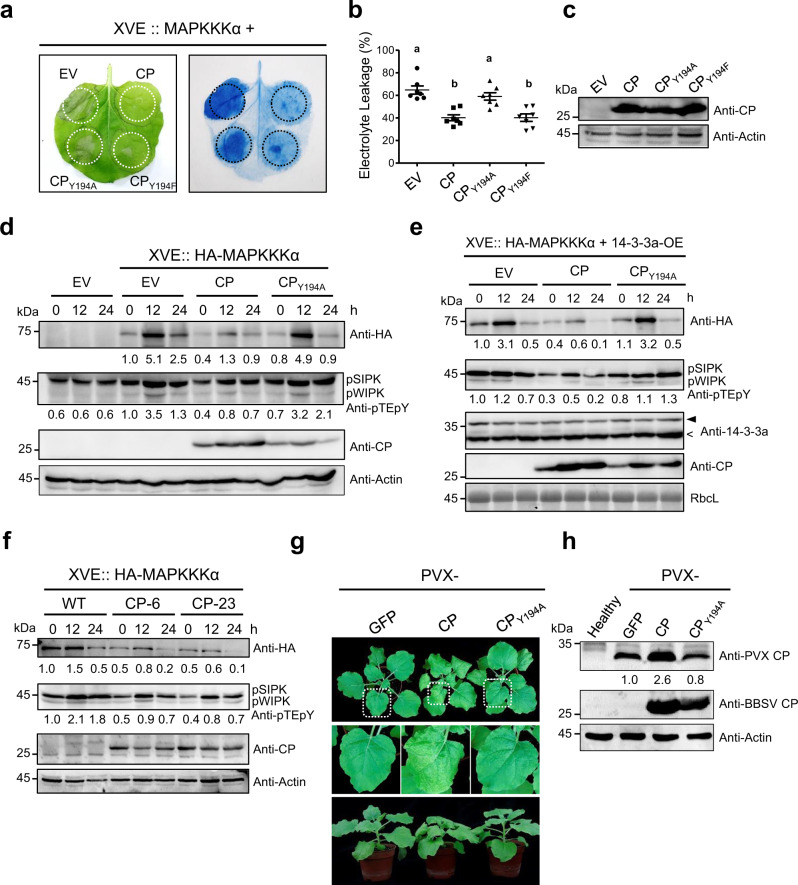
CP suppresses the MAPKKKα-mediated immune signaling by targeting Nb14-3-3a. **a** Interaction with 14-3-3a is required for CP to inhibit MAPKKKα-induced cell death. *Agrobacterium* carrying wild-type CP or its variants was co-infiltrated with *Agrobacterium* containing HA-MAPKKKα controlled by an estradiol-inducible system into different leaf regions. β-estradiol was used to induce protein expression 48 h after agroinfiltration. Leaves were stained by trypan blue and representative photographs were taken three days after estradiol treatment. **b** Quantification of cell death by measuring electrolyte leakage of leaf regions shown in (**a**). Error bars indicate ± SD of the mean (*n* = 6 biologically independent plants). Different letters in the chart denote statistically significant differences among different groups according to the one-way ANOVA analysis with Tukey’s multiple comparison test (*P* < 0.05). **c** Western blot analysis to confirm the expression of the CP or its variants with an anti-CP antibody. **d** CP suppresses MAPKKKα protein accumulation and MAPK activation in WT *N. benthamiana*. *Agrobacterium* carrying CP, CP_Y194A_, or empty vector (EV) control was co-infiltrated with *Agrobacterium* containing HA-MAPKKKα controlled by an estradiol-inducible system into leaves. 30 μM β-estradiol was infiltrated into the leaves 48 h after agroinfiltration. Samples were collected at 0, 12, 24 h after β-estradiol treatment and subjected to western blot analysis with different antibodies indicated on the right. Actin served as the loading control. **e** CP suppresses MAPKKKα protein accumulation and MAPK activation in *14-3-3a*-OE transgenic *N. benthamiana*. *Agrobacterium* carrying CP, CP_Y194A_ or empty vector was co-infiltrated with *Agrobacterium* containing HA-MAPKKKα controlled by an estradiol-inducible system into leaves. In total, 30 μM β-estradiol was applied to the leaves 48 h after agroinfiltration. Samples were collected at 0, 12, 24 h after β-estradiol treatment and subjected to western blot analysis with different antibodies indicated on the right. CBB-stained RbcL served as the loading control. **f** CP-transgenic *N. benthamiana* exhibited reduced accumulation of HA-MAPKKKα protein and MAPK phosphorylation compared with WT plants. *Agrobacterium* carrying HA-MAPKKKα controlled by an estradiol-inducible system was infiltrated into leaves. In all, 30 μM β-estradiol was applied to the leaves 48 h after agroinfiltration. Samples were collected at 0, 12, 24 h after β-estradiol treatment and subjected to western blot with different antibodies indicated on the right. Actin served as the loading control. For panels **c**–**f**, the experiments were repeated three times with similar results. **g** Symptom observation of engineered PVX-inoculated *N. benthamiana* plants. *Agrobacterium* carrying engineered PVX infection clones were infiltrated into two leaves of each plant. Representative photographs were taken at 6 dpi. **h** Western blot analysis of viral CP in the systemic leaves shown in panel g by using anti-PVX-CP or anti-BBSV CP antibody. Actin served as the loading control. The experiment was repeated three times with similar results.

To investigate whether CP suppressed MAPKKKα-mediated cell death by binding to 14-3-3a, wild-type or mutant CPs were co-expressed with estradiol-induced HA-MAPKKKα. Trypan blue staining and electrolyte-leakage assays showed that leaf tissues expressing CP and CP_Y194F_, but not CP_Y194A_, exhibited reduced cell death induced by MAPKKKα compared with the EV control (Fig. 7a, b). The expression of CP proteins was confirmed by western blot analysis (Fig. 7c). Since CP_Y194A_ barely interact with 14-3-3a (Fig. 6d–f), we conclude that inhibition of MAPKKKα-induced cell death by viral CP is dependent on its interaction with 14-3-3a.

To investigate the molecular basis underpinning the CP inhibition of MAPKKKα-induced cell death, we detected the levels of MAPKKKα proteins and activation of MAPKs (phosphorylation of SIPK and WIPK) at different time points after induction of MAPKKKα expression in the presence of CP or CP_Y194A_. Western blot analysis showed that, compared with EV control, the abundance of HA-MAPKKKα was decreased in the leaves expressing CP, but showed no obvious changes in the leaves expressing CP_Y194A_ (Fig. 7d). Consistently, the levels of SIPK and WIPK phosphorylation were also decreased in leaves expressing CP (Fig. 7d). To determine whether CP was able to impair the enhancement of 14-3-3a on MAPKKKα-mediated signaling, we conducted a similar assay in 14-3-3a-OE transgenic *N. benthamiana* plants. Levels of both MAPKKKα proteins and MAPKs phosphorylation were decreased in the leaves expressing CP (Fig. 7e). We also generated stable *N. benthamiana* transgenic lines overexpressing CP. Compared with the WT plants, two independent *N. benthamiana* plants stably overexpressing CP showed a decreased abundance of MAPKKKα and reduced phosphorylation of MAPKs after induction of MAPKKKα expression (Fig. 7f). Together, these results indicate that CP suppresses the activation of MAPKs by reducing the abundance of MAPKKKα.

It has been previously shown that the CP of another RNA virus, potato virus X (PVX) accumulate to a higher level in *NbMAPKKKα*-silenced *N. benthamiana* plants^49^. Therefore, we tested whether BBSV CP mediated reduction of MAPKKKα could lead to enhanced PVX infection. For this, we engineered BBSV CP or CP_Y194A_ into PVX-based vector and agroinfiltrated into *N. benthamiana*. The symptoms caused by PVX-BBSV-CP were more severe than that of either PVX-BBSV-CP_Y194A_ or PVX-GFP (Fig. 7g). Consistently, the accumulations of both PVX and BBSV CP in the systemic leaves of PVX-BBSV-CP-infected *N. benthamiana* were higher than that in PVX-BBSV-CP_Y194A_ or PVX-GFP-infected plants (Fig. 7h). These results further revealed that BBSV CP has the ability to inhibit MAPKKKα-mediated antiviral immunity.

To further explore the biological significance of the CP-14-3-3a interaction on MAPKKKα-mediated defense against BBSV infection, we generated BBSV mutant by replacing CP with CP_Y194A_ (BBSV_Y194A_) and inoculated them onto WT or *MAPKKKα-*KO plants. We observed cell-death phenotype on the leaves of WT plants inoculated with BBSV_Y194A_ at 2 dpi but not for the leaves inoculated with WT-BBSV (Supplementary Fig. 9a). In contrast, neither BBSV nor BBSV_Y194A_ induced cell-death phenotype on the inoculated leaves of *MAPKKKα-*KO plants (Supplementary Fig. 9a), indicating that the BBSV_Y194A_-induced cell death depends on the MAPKKKα. Consistent with this, leaves inoculated with BBSV_Y194A_ showed a lower level of CP accumulation compared with BBSV, and knocking out of the *NbMAPKKKα* gene partially rescues the accumulation level of BBSV_Y194A_ (Supplementary Fig. 9b). We did not observe the systemic infection of BBSV_Y194A_ in either WT or *MAPKKKα-*KO plants (Supplementary Fig. 9b, c), suggesting the Y194 in CP is critical for BBSV systemic infection. Since the Y194A mutation eliminates the interaction of CP with 14-3-3a, these results indicate that the interaction of CP with 14-3-3a is essential for suppressing MAPKKKα-mediated cell death during BBSV infection.

### CP competitively interferes with the MAPKKKα binding to 14-3-3a

**Fig. 8:**
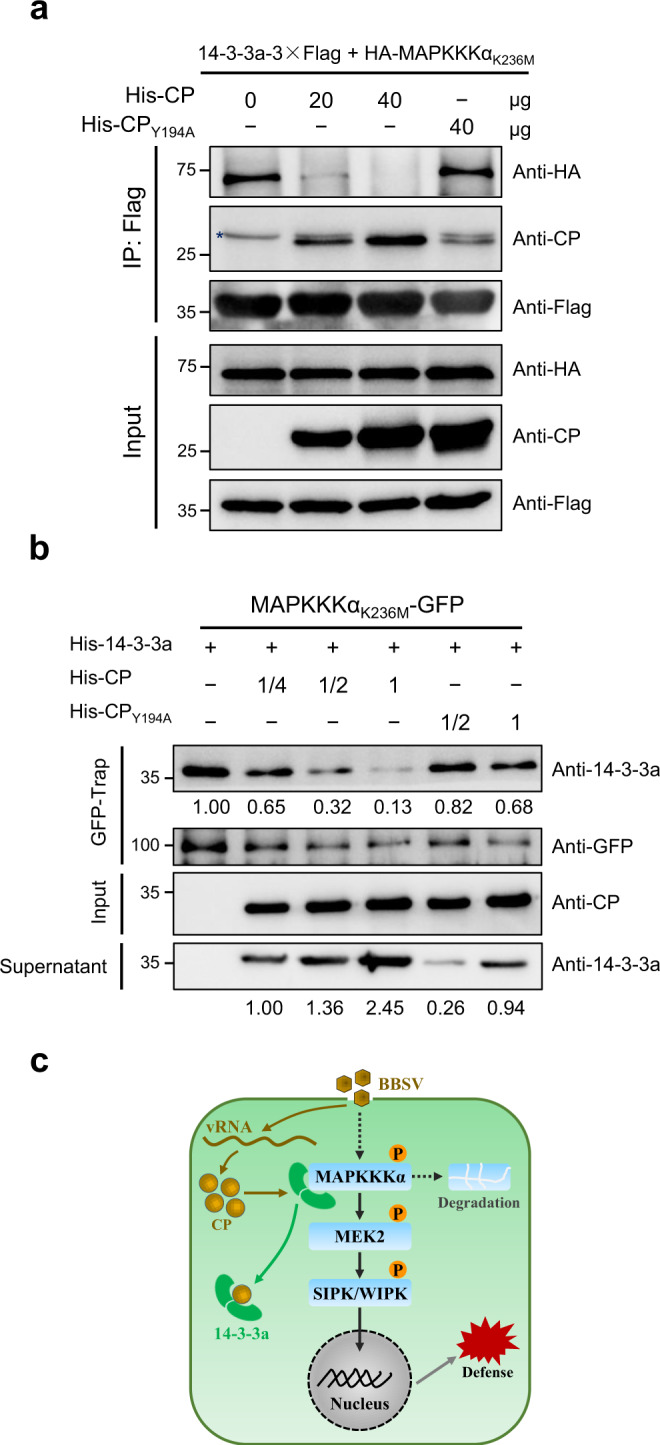
CP interferes with the MAPKKKα interaction with 14-3-3a in a dose-dependent manner. **a** Competitive Co-IP assay. His-CP and His-CP_Y194A_ were purified from *E. coli*. 14-3-3a-3×Flag and HA-MAPKKKα_K236M_ were co-expressed in *N. benthamiana* leaves and immunoprecipitated with anti-Flag beads. Anti-Flag beads with 14-3-3a-3×Flag and HA-MAPKKKα_K236M_ proteins was incubated with increasing amounts of His-CP protein (0, 20, and 40 μg) or His-CP_Y194A_ (40 μg). Input and IP proteins were analyzed by western blot with different antibodies indicated on the right. Asterisk indicates nonspecific bands. The experiment was repeated three times with similar results. **b** Competitive pull-down assay. MAPKKKα_K236M_-GFP was expressed in *N. benthamiana* leaves and immunoprecipitated with GFP-Trap agarose. After removing the supernatant, GFP-Trap agarose was incubated with *E. coli*-expressed His-14-3-3a followed by removal of the supernatant. GFP-Trap agarose was incubated with a serial dilution of His-CP or His-CP_Y194A_ (1, 1/2, 1/4). The resultant agarose was analyzed by western blot with different antibodies indicated on the right. The experiment was repeated three times with similar results. **c** A proposed model for CP-Nb14-3-3a-MAPKKKα functional module in plant–virus interaction. Virus infection leads to the activation of the MAPK cascade-mediated antiviral defense. In turn, viral CP disturbs the interaction between 14-3-3a and MAPKKKα, resulting in the degradation of MAPKKKα and the attenuation of antiviral response.

Given that both CP and MAPKKKα interact with 14-3-3a but CP does not interact with MAPKKKα (Figs. 3 and 4a, b and Supplementary Fig. 4), we wonder whether CP interferes with the interaction of MAPKKKα with 14-3-3a. To test this hypothesis, we performed a competitive Co-IP assay. *Agrobacterium* containing 14-3-3a-3×Flag and HA-MAPKKKα_K236M_ expression cassette were co-infiltrated into the *N. benthamiana* leaves. Leaf homogenates were prepared and mixed with *E. coli*-expressed His-tagged CP proteins. After incubation for 40 min, the homogenates were precipitated with anti-Flag beads, and the eluates were then examined by western blot. The results showed that the association between 14-3-3a-3×Flag and HA-MAPKKKα_K236M_ was reduced with the addition of increasing amounts of *E. coli*-expressed His-tagged CP (His-CP). However, CP_Y194A_ mutant protein, which barely interacts with 14-3-3a, had little effect on the interaction of 14-3-3a-3×Flag with HA-MAPKKKα_K236M_ (Fig. 8a). Furthermore, we performed GFP competitive pull-down assay. Because *E. coli*-expressed MAPKKKα was insoluble, GFP-fused MAPKKKα_K236M_ was expressed in *N. benthamiana* followed by purification with GFP-Trap beads. An excessive amount of *E. coli*-expressed His-14-3-3a was used to saturate the beads, and the supernatant was removed. With the addition of an increasing amount of *E. coli*-expressed His-CP to the above GFP-Trap beads, more and more His-14-3-3a was pulled off from the beads concomitant with increased levels of His-14-3-3a in the supernatant (Fig. 8b). These data indicate that CP competitively interferes the interaction between MAPKKKα and 14-3-3a, leading to a decrease in the abundance of MAPKKKα (Fig. 8c).

### Perturbation of 14-3-3a-MAPKKKα functional module by the CP may be a common strategy employed by necroviruses

**Fig. 9:**
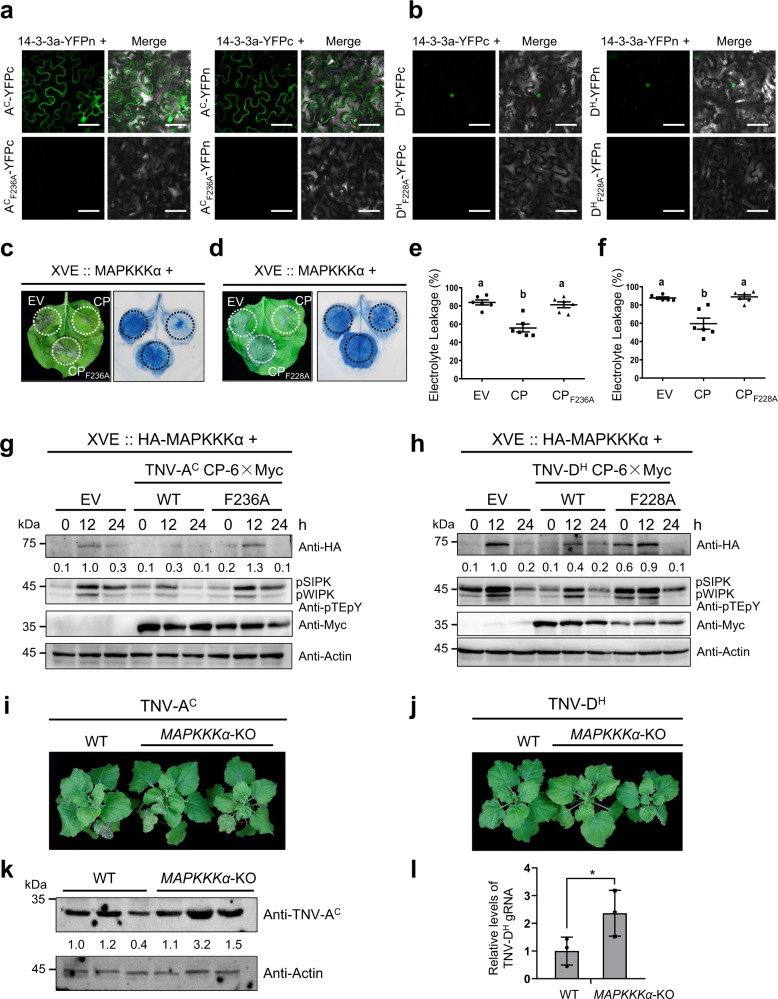
Perturbation of Nb14-3-3a-MAPKKKα functional module by the CP is a common strategy employed by necroviruses. **a**, **b** BiFC analysis of the interaction between CP of TNV-A^C^ (**a**) or TNV-D^H^ (**b**) and 14-3-3a. The indicated combinations of different proteins were transiently co-expressed in *N. benthamiana* leaves. Confocal analysis was performed at 3 dpi. The experiments were repeated three times and representative results are shown. Scale bars = 50 µm. **c**, **d** CP inhibits MAPKKKα-induced cell death. *Agrobacterium* carrying wild-type TNV-A^C^ (**c**) or TNV-D^H^ (**d**) CP or their variants was co-infiltrated with *Agrobacterium* containing HA-MAPKKKα controlled by an estradiol-inducible system into different leaf regions. Empty vector (EV) served as the negative control. β-estradiol was applied 48 h after agroinfiltration. Leaves were stained by trypan blue and representative photographs were taken 3 days after estradiol treatment. **e**, **f** Quantification of cell death by measuring electrolyte leakage of leaf regions shown in (**c**) and (**d**). Error bars indicate ± SD of the mean (*n* = 6 biologically independent plants). Different letters in the chart denote statistically significant differences among different groups according to the one-way ANOVA analysis with Tukey’s multiple comparison test (*P* < 0.05). **g**, **h** CP affects MAPKKKα protein stability and MAPK activation in non-transgenic *N. benthamiana*. *Agrobacterium* carrying CP or CP_Y194A_ was co-infiltrated with *Agrobacterium* containing HA-MAPKKKα controlled by an estradiol-inducible system into the leaves. Empty vector (EV) served as the negative control. In all, 30 μM β-estradiol was infiltrated into the leaves 48 h after agroinfiltration. Samples were collected at 0, 12, 24 h after β-estradiol treatment and subjected to western blot with different antibodies indicated on the right. Actin served as the loading control. The experiments were repeated three times with similar results. **i**, **j** Phenotype observation of TNV-A^C^ (**i**) or TNV-D^H^ (**j**) -infected *MAPKKKα*-KO plants. *N. benthamiana* leaves were inoculated with 100 ng TNV-A^C^ virions. Representative photographs were taken at 9 dpi. **k** Western blot analysis of TNV-A^C^ CP levels in the plants shown in panel **i** using an anti-TNV-A^C^ antibody. Actin served as the loading control. The experiment was repeated three times with similar results. **l** RT-qPCR analysis of TNV-D^H^ genomic RNA levels in the plants shown in panel **j**. Values represent ± SD of the mean (*n* = 6 biologically independent plants). An asterisk indicates the significant difference based on one-sided Student’s *t* test (*P = 0.0357).

Sequence alignments showed that the 14-3-3a binding-like motif is conserved in the CPs of alphanecrovirus and betanecrovirus (Supplementary Fig. 10), suggesting that the perturbation of 14-3-3a-MAPKKKα functional module by CP may also be employed by other necroviruses. To test this hypothesis, the conserved 14-3-3a binding site F^236^ in the CP of Tobacco necrosis virus-A (TNV-A^C^, alphanecrovirus) and F^228^ in the CP of TNV-D^H^ (betanecrovirus) were mutated to alanine. BiFC assay showed that co-expression of 14-3-3a with wild-type TNV-A^C^ and TNV-D^H^ CPs, but not mutant CPs, resulted in the reconstitution of fluorescence signals (Fig. 9a, b). Expression of the test proteins was confirmed by western blot analysis (Supplementary Fig. 3f). These results indicate that both TNV-A^C^ and TNV-D^H^ CPs interact with 14-3-3a, and the amino acids F^236^ or F^228^ within the CPs are crucial for their interaction with 14-3-3a.

To examine the effect of TNV-A^C^ and TNV-D^H^ CPs on MAPKKKα-induced cell death, wild-type or mutant CPs was co-expressed with HA-MAPKKKα controlled by an estradiol-inducible system. Trypan blue staining and the electrolyte-leakage assays showed that compared with the EV control, MAPKKKα-induced cell death was significantly decreased in leaf tissues expressing wild-type CPs, but not mutant CPs (Fig. 9c–f). Consistently, the levels of MAPKKKα proteins and downstream MAPK phosphorylation level were decreased in the leaves expressing wild-type CPs, but not mutant CPs, compared with the EV control (Fig. 9g, h).

We also investigated whether 14-3-3a and MAPKKKα play antiviral roles in TNV-A^C^ and TNV-D^H^ infection. *14-3-3a*-OE and *MAPKKKα-*KO plants were inoculated with TNV-A^C^ or TNV-D^H^, respectively. TNV-A^C^ and TNV-D^H^ caused more severe symptoms in *MAPKKKα-*KO plants than WT plants (Fig. 9i, j). Western blot and RT-qPCR analysis showed that both TNV-A^C^ and TNV-D^H^ accumulation was increased in *MAPKKKα-*KO plants (Fig. 9k, l) and significantly decreased in *14-3-3a*-OE plants (Supplementary Fig. 11). These findings indicate that other necroviruses may utilize the same strategy as that of BBSV to counteract MAPKKKα-mediated antiviral immunity.

## Discussion

MAPK cascades play a central role in defense signaling against pathogens^20^. In animals, both DNA and RNA viruses are known to induce the MAPK cascades^50,51^. They utilize and fine-tune MAPK cascades to promote viral replication^52,53^. However, the relationship between MAPK cascades and plant viruses remains understudied. Here, we show that MAPKKKα plays an antiviral role. Our findings showed that the MAPK cascade is activated during the early stage of BBSV infection as a defense mechanism. Consistent with this, downregulation of MAPKKKα, as well as the downstream MAPKs, enhanced the accumulation of BBSV. Likewise, MAPK cascades are also reported to be activated in tomato yellow leaf curl China virus (TYLCCNV)-infected *N. benthamiana*^36^ and TMV-infected tobacco plants expressing R protein N^28^. Knockout of NbSIPKK and NbMPK4 also enhances TYLCCNV infection and the suppression of NtMEK2-SIPK/WIPK cascade compromises *N*-mediated resistance against TMV. These studies, together with the data shown here indicate the negative effect of MAPK cascades in plant–virus infection. Our time-course analysis of the *NbMAPKKKα* mRNA levels during BBSV infection showed no significant change in *NbMAPKKKα* expression (Supplementary Fig. 12a), consistent with the RNA-seq data (Supplementary data 4). Although phosphorylation levels of SIPK and WIPK were significantly increased in response to BBSV infection (Fig. 1h, i), the protein levels of SIPK and WIPK were also not altered during BBSV infection (Supplementary Fig. 12c). These results suggest that BBSV-induced activation of MAPK signaling pathway is through modulation of the phosphorylation status of MAPKKKα-MEK2-SIPK/WIPK components but not through the increased protein expression.

Although it has been reported that MAPK cascades play an important role in plant defense against a variety of pathogens^18^, the mechanism underlying MAPK-mediated defense against plant–virus infection remains largely unclear. Here, we performed RNA-seq analysis to reveal that the expression of a large number of genes were transcriptionally regulated in response to BBSV infection, and some of these genes are specifically regulated through MAPKKKα (Fig. 2). For example, *PR1A* (a salicylic acid (SA)-responsive gene), *ERF1B* (a transcription factor that is involved in ethylene signaling), and *HIR1* (specifically associated with the HR cell death) are all reported to be regulated during plant defense against virus^40,54,55^. Moreover, the SA pathway, ethylene pathway, and HR cell death are reported to cross-talk with each other and also with MAPK signaling pathway^26,56–58^. Hence, our results demonstrated that MAPKKKα plays an antiviral role by activating MAPK signaling and inducing downstream defense-related gene expression, which provides a global insight into the mechanisms underlying the MAPKKKα-mediated antiviral immunity. Nevertheless, more details on how these genes perceive the MAPK signaling and induce antiviral response needs further investigation. Besides, how viruses activate MAPK cascades is a remaining gap to be addressed in the future. It is likely that intracellular or extracellular receptors recognize some conserved structures such as the virions, viral ribonucleoprotein complexes, or viral-derived dsRNAs to initiate innate immune signaling^59^.

Although the activation of MAPK signaling has some effect on BBSV infection, it is not sufficient to block virus infection suggesting that BBSV may have evolved a strategy to suppress MAPK signaling. Our data showed that BBSV CP is responsible for counteracting MAPKKKα-mediated antiviral defense. Interestingly, unlike *Pseudomonas* bacterial effectors HopF2, HopAI1, and AvrRpt2, or the βC1 encoded by a satellite associated with a DNA virus that directly interacts with MAPKs and inactivates them to compromise defense responses^33–36^, BBSV CP does not interact with MAPKKKα. Instead, it interacts with 14-3-3a to modulate MAPKKKα-mediated antiviral immunity.

The 14-3-3 proteins are small acidic proteins (∼30 kDa) that are highly conserved in eukaryotes^60,61^ 14-3-3 proteins play important roles in signal transduction pathways to regulate diverse biological processes, such as cell division, primary metabolism, biotic and abiotic stress responses by interacting with various proteins in both plants and animals^62–64^. Accumulating evidence indicates that 14-3-3 proteins are involved in plant immunity^41,42,65^. 14-3-3 proteins interact with various signaling components of the plant immune system and modulate their activities, such as NADPH oxidase NtrbohD in *N. tabacum*^66^, plasma membrane H^+^-ATPase in maize and *Arabidopsis*^67^, R protein RPW8.2 in *Arabidopsis*^68^, and MAPKKKα in tomato^69^. Although these studies unveil the important roles of 14-3-3 proteins in plant defense to nonviral pathogens, their roles in plant virus infection remain unexplored. Our findings described here, for the first time, revealed that 14-3-3a protein plays an antiviral role. Although we cannot rule out the possibility that other 14-3-3 isoforms may also participate in the plant immune response, our results indicate that 14-3-3a is at least one of the key regulators.

In animals, 14-3-3 interacts with and activates two MAPKKKs, Raf-1 and MEKK3^70,71^. In plants, ZmMPK6 is reported to interact with a maize 14-3-3 protein GF14-6^72^. SIMAPKKKα is reported to interact with a tomato 14-3-3 protein TFT7^69^. These results prompted us to test whether NbMAPKKKα could interact with 14-3-3a in *N. benthamiana*. Our results confirmed their interaction and revealed that 14-3-3a is required for NbMAPKKKα-mediated cell death in *N. benthamiana*, which is in accordance with the positive regulation of SIMAPKKKα by TFT7 in tomato^69^. Our results also showed that 14-3-3a could protect the MAPKKKα from ubiquitination-mediated degradation (Fig. 4i). Genetic analysis using *MAPKKKα*-KO plants indicated that 14-3-3a participate in MAPKKKα-mediated defense against BBSV infection. As a counter-defensive strategy, BBSV uses its CP to suppress MAPKKKα-mediated defense by directly interacting with 14-3-3a that reduces the MAPKKKα accumulation. Previous studies also showed that downregulation of MAPKKKα expression increases the accumulation of PVX in *N. benthamiana*^49^. Interestingly, expression of BBSV CP in the PVX infectious clone resulted in enhanced accumulation of PVX and symptoms, further supporting the BBSV CP suppression of MAPKKKα-mediated plant defense. We also performed a time-course analysis of the *Nb14-3-3a* expression during BBSV infection and found no significant changes in the *Nb14-3-3a* expression levels (Supplementary Fig. 12b, c), consistent with the RNA-seq data (Supplementary Data 5). Considering that the Nb14-3-3a-mediated antiviral defense depends on the MAPKKKα (Fig. 5) and the relatively high abundance of the endogenous plant 14-3-3 proteins (Supplementary Fig. 12c), we speculate that the endogenous level of 14-3-3 might be sufficient to activate MAPK signaling.

Previous studies indicated that bacterial pathogen effectors target 14-3-3 proteins. For example, the XopQ effector from *Xanthomonas oryzae* pv. *oryzae* interacts with several rice 14-3-3 proteins to regulate immunity^73^. The effectors XopQ, XopE1, XopE2, and XopO from *Xanthomonas euvesicatoria* interact with multiple tomato 14-3-3 proteins to suppress ETI^74,75^. The effector XopN from *Xanthomonas campestris* pv. *vesicatoria* interacts with a tomato atypical receptor-like kinase 1 (TARK1) and TFT1 to suppress PTI^76,77^. Here, for the first time, we demonstrate that 14-3-3a is targeted by a viral CP effector. Based on our findings, we propose a model illustrating the MAPKKKα-mediated defense against viruses and how viral CP counteracts the MAPKKKα-mediated immunity (Fig. 8c). Upon perception of viral infection, MAPKKKα is activated by some unknown components and 14-3-3a binds to and enhances the stability of MAPKKKα, leading to the activation of MAPK signaling and concomitant induction of plant defense-related genes, which may contribute to immunity against virus infection. To counteract the MAPKKKα-mediated antiviral immunity, CP is translated from viral RNA (vRNA) and increased amount of CP accumulates during the viral infection, it interacts with 14-3-3a and disrupts the 14-3-3a-MAPKKKα module. The released MAPKKKα is degraded by the 26S proteasome, leading to the impairment of MAPKKKα-mediated antiviral resistance (Fig. 8c). Our findings provide novel insights towards a better understanding of defense and counter-defense during plant–virus interplay. Our results also show that CPs of other necroviruses interfere with 14-3-3a-MAPKKKα immune signaling module as a counter-defensive strategy to promote viral infection. Moreover, we also noted that CPs of numerous viruses in the family *Tombusviridae* contain the 14-3-3-binding-like motif^78^, implying a general mechanism employed by the viruses in the *Tombusviridae* to subvert plant immunity.

In summary, our findings described here deepen our understanding of the MAPKKKα-mediated defense against viruses and reveal a novel mechanism for the viral CP in counteracting MAPKKKα-mediated antiviral immunity.

## Methods

### Plant materials and growth conditions

*Nicotiana benthamiana* plants were grown in a growth chamber at 23–24 °C with a 15-h-light/9-h-dark cycle.

### Generation of transgenic *N*. *benthamiana* plants

*14-3-3a*-OE and *14-3-3a*-KD transgenic *N. benthamiana* plants were generated by *Agrobacterium*-mediated transformation with binary vectors 14-3-3a-3×Flag and 14-3-3a-RNAi, respectively. *MAPKKK*α*-KO* transgenic *N. benthamiana* plants were generated by *Agrobacterium*-mediated transformation with binary vector BGK01-MAPKKKα. Single guide RNA targeting open reading frame of MAPKKKα was designed using CRISPR-P 2.0 design tool (http://crispr.hzau.edu.cn/CRISPR2/). Plasmids were transformed into *A*. *tumefaciens* strain EHA105. Leaf disc transformation was performed to generate the transgenic *N*. *benthamiana* plants^79^. Western blots with anti-14-3-3a antibodies were performed to screen the positive *14-3-3a*-OE and *14-3-3a*-KD transgenic plants. To screen the positive *NbMAPKKKα* knockout plants, DNA fragment corresponding to the gRNA-targeted DNA sequence regions was amplified from genomic DNA of transgenic plants using corresponding primers listed in Supplementary Data 6, and DNA sequencing was carried out to examine the insertion or deletion at the target site.

### Construction of plasmids

BBSV derivatives used in this study were generated from pCB301-BBSV^80^. The sequence of sfGFP11 and linker was amplified using synthetic oligos and introduced into N-terminal of CP in BBSV infectious clone using reverse PCR to generate BBSV-sfGFP_11_. Single amino acid mutation within the BBSV CP was constructed by using the QuickChange Site-Directed Mutagenesis Kit (Agilent Technologies, Santa Clara, CA, USA) according to the manufacturer’s instructions.

For transient expression in plants, plasmid pMDC32-CP_Y194A_ and pMDC32-CP_Y194F_ were constructed by amplifying the CP fragments from infectious clones of mutant BBSV containing Y194A or Y194F respectively and recombined into *Kpn* I and *Spe* I-digested pMDC32 plasmid^81^ using the Seamless Assembly Cloning Kit (Clone Smarter). Nb14-3-3a was amplified from the cDNA of *N. benthamiana* and cloned into pMD19-T vector (Takara). The obtained plasmid was used for site-directed mutagenesis by using a QuickChange Site-Directed Mutagenesis Kit. The DNA fragment encoding wild-type or mutant Nb14-3-3a was amplified and cloned into pMDC32-3×Flag using the Seamless Assembly Cloning Kit (Clone Smarter) to generate 14-3-3a-3×Flag, 14-3-3a_3E_-3×Flag, and 14-3-3a_QQR_-3×Flag respectively. The construction of the plasmid pMDC32-3×Flag was described previously^82^. For the construction of HA-MAPKKKα_K236M_, NbMAPKKKα was amplified from the cDNA of *N. benthamiana* and cloned into the pGD-3×HA vector, an updated version of pGD vector^83^ that was engineered with an N-terminal triple HA epitope. The resultant plasmid was used for site-directed mutagenesis using a QuickChange Site-Directed Mutagenesis Kit. To generate XVE::HA-MAPKKKα, MAPKKKα with a N-terminal triple HA epitope was amplified and recombined into pDONR207 vector (Invitrogen) by BP recombination followed by LR recombination into destination vector SPDK2130 under the estradiol-inducible system^84^. The other plasmids including pMDC32-CP, CP-3×Flag, and GFP-3×Flag were described previously^80^.

For TRV-mediated gene silencing, the cDNA fragments corresponding to the putative *NbMEK2*, *NbSIPK* and *NbWIPK* were amplified using cDNA of *N. benthamiana* and cloned into pYL156^85^.

For BiFC assay, the 14-3-3a gene and its mutants (14-3-3a_3E_ and 14-3-3a_QQR_), CP wild-type gene and its mutants (CP_1-187_, CP_46-232_, CP_Y194A_ and CP_Y194F_), and MAPKKKα_K236M_ were amplified respectively and cloned into binary vectors pSPYNE-35S or pSPYCE-35S^86^.

To generate 14-3-3a-RNAi and MAPKKKα-RNAi vector, 300 nucleotides from 14-3-3a cDNA and 1-322 nucleotides from MAPKKKα cDNA were amplified and recombined into pDONR207 vector (Invitrogen) by BP recombination respectively. The interference fragments were then introduced into the destination vector pHELLSGATE 8^87^ via LR reaction to generate RNAi expression vector.

For prokaryotic expression experiments, *Nb14-3-3a* gene was amplified and cloned into *Eco*R I-digested pGEX-KG plasmid to generate GST-14-3-3a. His-CP, His-CP_Y194A_, and His-CP_Y194F_ were constructed by amplifying the CP fragments from the infectious clones of wild-type or mutant BBSV and ligated into pET30a (+) at the *Bam*HI and *Sal*I restriction sites. The construction of GFP-His was described previously^80^.

For PVX infection assay, BBSV CP and CP_Y194A_ were cloned into the pND108 vector^88^ at the *Xho*I and *Apa*I sites to yield PVX-CP and PVX-CP_Y194A_.

The primers used for constructing these plasmids are listed in Supplementary Data 6 and DNA sequencing was performed to confirm the correctness of all the plasmids.

### Agroinfiltration and viral inoculation

All plasmids were transformed into *Agrobacterium tumefaciens* strains GV3101 or EHA105 by freeze–thaw transformation^89^. After being cultured in LB medium containing 25 μg/mL kanamycin and 100 μg/mL rifampicin at 28 °C for 14–16 h, *Agrobacterium* cells were harvested and resuspended in infiltration media (10 mM MgCl_2_, 150 μM acetosyringone, and 10 mM MES (pH 5.6)) for 2–4 h at room temperature followed by infiltration into four-week-old *N. benthamiana* leaves using needle-free syringes. For co-expression assays, *Agrobacterium* containing different constructs were mixed in a 1:1 or 1:1:1 ratio with a final OD_600_ = 0.3. For the XVE::HA-MAPKKKα (wild-type or K236M) expression, 30 µM 17-β-estradiol were infiltrated into the *N. benthamiana* leaves 48 h after agroinfiltration.

For viral infection analysis, 300 ng of BBSV or TNV-A^C^ virions mixed with FES inoculation buffer (0.1 M glycine, 0.06 M dipotassium phosphate, 1% sodium pyrophosphate decahydrate, 1% bentonite, 1% celite, pH 8.5) at a 1:1 ratio followed by mechanical inoculation onto 4–5 leaf stage *N. benthamiana* plants. RNA transcripts of TNV-D^H^ were inoculated as described previously^82^. The *Sma*I-linearized plasmid A172S^90^ (200 ng) were used as templates for in vitro transcription with T7 RNA polymerase (Promega). Freshly prepared in vitro transcripts were analyzed by 1% (wt/vol) agarose gel electrophoresis to evaluate the quality of the RNA transcripts. The RNA transcripts were then mixed with an equal volume of FES inoculation buffer at a 1:1 ratio followed by mechanical inoculation onto 4–5 leaf stage *N. benthamiana* plants.

### RNA-Seq analysis

The leaf samples from Mock- and BBSV-inoculated plants were collected at the indicated dpi. Three leaves from different plants were collected as one biological replicate, and three biological replicates were used for each group. Total RNAs were extracted using TRIzol reagent (Invitrogen). After quantification and qualification, total RNAs were pooled for cDNA library construction. cDNA library preparation and sequencing were carried out on an Illumina NovaSeq 6000 platform with 150-bp pair-end reads in Novogene (Beijing, China). Total reads were mapped to the *Nicotiana benthamiana* transcriptome (http://sefapps02.qut.edu.au/downloads/Nbv0.5.genome.fa.gz) using Hisat2 (v2.0.5), and the mapped reads of each sample were assembled by StringTie (v1.3.3b) in a reference-based approach. The expression level of each gene was calculated based on expected number of Fragments Per Kilobase of transcript sequence per Million’s base pairs sequenced (FPKM). Differential expression analysis of two groups was performed using the DESeq2 R package (1.20.0). The resulting *P* values were adjusted using Benjamini and Hochberg’s approach for controlling the false discovery rate (*P*adj). *P*adj < 0.05 and |log2(foldchange)| >1 were set as the threshold for significantly differential expression.

### Mass spectrometry analysis

Silver-stained protein band as indicated by the rectangle in Fig. 3c was excised from SDS-PAGE gels, the gel band was destained with Sigma Proteosilver kit (PROTSIL1, Sigma), and then reduced with 10 mM DTT, alkylated with 55 mM iodoacetamide, and digested with trypsin (pH 8.5) at 37 °C for 12 h. The digested peptides were separated by nanoscale C18 reverse-phase liquid chromatography (Waters), and then electro-sprayed into a Q-Exactive high-resolution mass spectrometer (Thermo Fisher Scientific) at the Mass Spectrometry Facility of China Agricultural University. Protein identification was performed by searching against the protein database of either the National Center for Biotechnology Information (NCBI) or the *N*. *benthamiana* sequence v1.0.1 proteome (ftp://ftp.solgenomics.net/genomes/Nicotiana_benthamiana) using Mascot Server (version 2.5.1, Matrix Science).

### BiFC assay

*Agrobacterium* harboring various BiFC constructs were mixed with a final OD_600_ = 0.3 and co-infiltrated into 4-week-old *N. benthamiana* leaves. At 3 dpi, the leaf samples were observed under a Zeiss LSM880 confocal microscope. YFP signals were excited at 514 nm.

### Co-immunoprecipitation (Co-IP)

Co-IP was performed according to previously described methods with minor modifications^91^. *N. benthamiana* leaves were co-infiltrated with the mixture of *Agrobacterium* containing various constructs. At 3 dpi, leaves were harvested and ground in liquid nitrogen followed by mixing with two volumes of freshly prepared protein extraction buffer [50 mM Tris-HCl (pH 7.5), 150 mM NaCl, 1 mM EDTA, 10% (v/v) glycerol, 2% (w/v) polyvinylpyrrolidone, 10 mM dithiothreitol (DTT), 1× protease inhibitor cocktail, and 0.5% (v/v) Triton X-100]. After incubation on ice for 40 min, the crude extract was centrifugated at 17,000 × *g* for 30 min and the supernatant was incubated with anti-Flag M2 Affinity Gel (Sigma-Aldrich) at 4 °C for 4 h. The beads were washed three times with IP buffer [50 mM Tris-HCl (pH 7.5), 150 mM NaCl, 1 mM EDTA, 10% (v/v) glycerol, and 0.1% (v/v) Triton X-100] and then analyzed by western blot with anti-Flag (1:5000, Sigma, Cat. No. F1804) and anti-CP (1:2000, produced by Beijing Protein Innovation Co., Ltd) antibodies.

### Expression and purification of recombinant proteins

Protein purification was performed as described previously^82^. Expression constructs were transformed into *E. coli* strains BL21 or Rosetta. Cultures were grown at 37 °C until OD_600_ = 0.8, and protein expression was induced by 0.2 mM isopropyl-β-D-thiogalactopyranoside (IPTG) at 18 °C overnight. Cells were then collected and subjected to protein purification using Glutathione Sepharose^TM^ 4 Fast Flow (GE Healthcare) or Ni-NTA agarose (QIAGEN) according to the manufacturer’s instructions.

### GST pull-down assay

GST pull-down assay was conducted as described previously with minor modifications^80^. About 20 μg of purified GST-14-3-3a or GST proteins were incubated with 15 μL Glutathione Sepharose^TM^ 4 Fast Flow (GE Healthcare) in 1 mL binding buffer (50 mM Tris-HCl (pH 7.5), 250 mM NaCl, 5 mM DTT, 0.2% (v/v) glycerol, and 0.6% (v/v) Triton X-100) for 1.5 h at room temperature. The supernatant was removed after centrifugation, and 10 μg of His-tagged proteins in 1 mL of binding buffer were incubated with the GST beads at room temperature for 2 h. The GST beads were washed six times with washing buffer (50 mM Tris-HCl (pH 7.5), 500 mM NaCl, 0.2% (v/v) glycerol, and 0.6% (v/v) Triton X-100) and detected by western blot with anti-His (1:5000, EASYBIO, Cat. No. BE7001) and anti-GST antibodies (1:5000, Genscript, Cat. No. A00866).

### Trypan blue staining

Trypan blue staining was performed as previously described with minor modifications^92^. The infiltrated leaves were pretreated with ethanol for 3 min, and then combined with trypan blue staining solution prepared in 40 mL of ethanol, 10 mL of lactic acid, 10 mL of water phenol, 10 mL of glycerol, and 10 mL of sterile water with 15 mg of Trypan blue. The samples were placed in boiling water for 10 min and incubated at room temperature for 8 h. The samples were destained using chloral hydrate (2.5 g/mL sterile water) followed by photography.

### Electrolyte-leakage assay

The electrolyte-leakage assay was performed as described previously with minor modifications^93^. Two leaf discs (9 mm in diameter) were collected from infiltrated areas of six to eight plants and floated in 15-mL tubes containing 10 mL of MilliQ-water for 1 h at 25 °C with shaking at 165 rpm. Conductivity was measured using a DDS-12DW conductivity meter (BANTE) to obtain S1 value. The samples were placed in boiling water for 45 min, followed by shaking at 25 °C for 1 h, and measured to obtain the S2 value. The ratio of S1 to S2 was calculated as electrolyte leakage.

### RNA extraction and reverse transcription-quantitative real-time PCR (RT-qPCR)

Total RNA was extracted using the TRIzol Reagent (Invitrogen) according to the manufacturer’s instructions. RT-qPCR was performed as previously reported^94^. Briefly, 3 μg of total RNA was treated with DNase I (Takara), and cDNA was reversely transcribed with M-MLV reverse transcriptase (Promega). qPCR analysis was performed with CFX Manger (Bio-Rad) using 2 × SsoFast EvaGreen Supermix (Bio-Rad). *Elongation factor 1α (EF-1α)* gene served as the internal control. All primer pairs used for RT-qPCR analyses are listed in Supplementary Data 6.

### MAPK activation assay

MAPK activation assay was performed as previously described with minor modifications^95^. After agroinfiltration and induction of MAPKKKα expression as described above, total protein was extracted in protein extraction buffer [50 mM Tris-HCl (pH 7.5), 150 mM of NaCl, 0.5% Triton X-100, 1% (v/v) protease inhibitor cocktail (Roche), 1 mM of Na_3_VO_4_, 1 mM of NaF, and 20 mM of β-glycerophosphate), and detected by western blot with anti-Phospho-p44/42 MAPK antibody (pTEpY) (1:2000, Cell Signaling Technology, Cat. No. 4370).

### Competitive Co-IP assay

The leaves infiltrated with 14-3-3a-3×Flag and HA-MAPKKKα_K236M_ were harvested at 3 dpi. Total protein was extracted in extraction buffer (50 mM Tris-HCl (pH 7.5), 150 mM NaCl, 1 mM EDTA, 10% (v/v) glycerol, 2% (w/v) polyvinylpyrrolidone, 10 mM dithiothreitol (DTT), 1 × protease inhibitor cocktail, and 0.5% (v/v) Triton X-100) on ice for 30 min, followed by centrifugation at 17,000 × *g* for 30 min. Then increasing amounts of purified His-CP (20 μg, 40 μg) or His-CP_Y194A_ (40 μg) were added to the supernatant and incubated with anti-Flag M2 Affinity Gel (Sigma-Aldrich). The precipitates were washed three times with IP buffer at 4 °C and analyzed by western blot with anti-Flag (1:5000, Sigma, Cat. No. F1804), anti-CP (1:2000, produced by Beijing Protein Innovation Co., Ltd) and anti-HA antibodies (1:5000, EASYBIO, Cat. No. BE7001).

### Competitive pull-down assay

A competitive pull-down assay was conducted as previously described with minor modifications^96^. MAPKKKα_K236M_-GFP extracts were immunoprecipitated by 20 μL GFP-Trap beads (ChromoTek, German) as described in the Co-IP part. 100 μg *E. coli*-expressed His-14-3-3a was added in 1 mL binding buffer [50 mM Tris-HCl (pH 7.5), 250 mM NaCl, 5 mM DTT, 1 mM PMSF, and 1× protease inhibitor cocktail] and incubated at 4 °C for 2 h. After two washes with washing buffer (50 mM Tris-HCl (pH 7.5), 250 mM NaCl, 5 mM DTT, 1 mM PMSF, 1× protease inhibitor cocktail and 0.2% (v/v) Triton X-100), 80 μg, 40 μg, 20 μg His-CP or 80 μg, 40 μg His-CP_Y194A_ was added to the 1 mL corresponding samples and incubated at 4 °C for additional 1 h. After three washes with washing buffer, samples were analyzed by western blot with anti-GFP (1:5000, MBL, Cat. No. 598), anti-CP (1:2000, produced by Beijing Protein Innovation Co., Ltd) and anti-14-3-3a antibodies (1:1000, produced by Beijing Protein Innovation Co., Ltd).

### Reporting summary

Further information on research design is available in the Nature Research Reporting Summary linked to this article.

## Supplementary information

Supplementary Information

Description of Additional Supplementary Files

Supplementary Data 1

Supplementary Data 2

Supplementary Data 3

Supplementary Data 4

Supplementary Data 5

Supplementary Data 6

Reporting Summary

## Data Availability

Data supporting the findings of this work are available within the paper and its Supplementary Information files, or from the corresponding author upon request. The raw RNA-seq data are available in the NCBI database under accession code PRJNA767174. Source data are provided with this paper.

## Source data

Source Data
