# Supplementary material for: Coat proteins of necroviruses target 14-3-3a to subvert MAPKKKα-mediated antiviral immunity in plants
Source: Nat Commun. 2022 Feb 7;13:716. doi: 10.1038/s41467-022-28395-5 (PMC8821596; doi:10.1038/s41467-022-28395-5)
Supplement: Supplementary file 1 — Supplementary Information [file 41467_2022_28395_MOESM1_ESM.pdf]

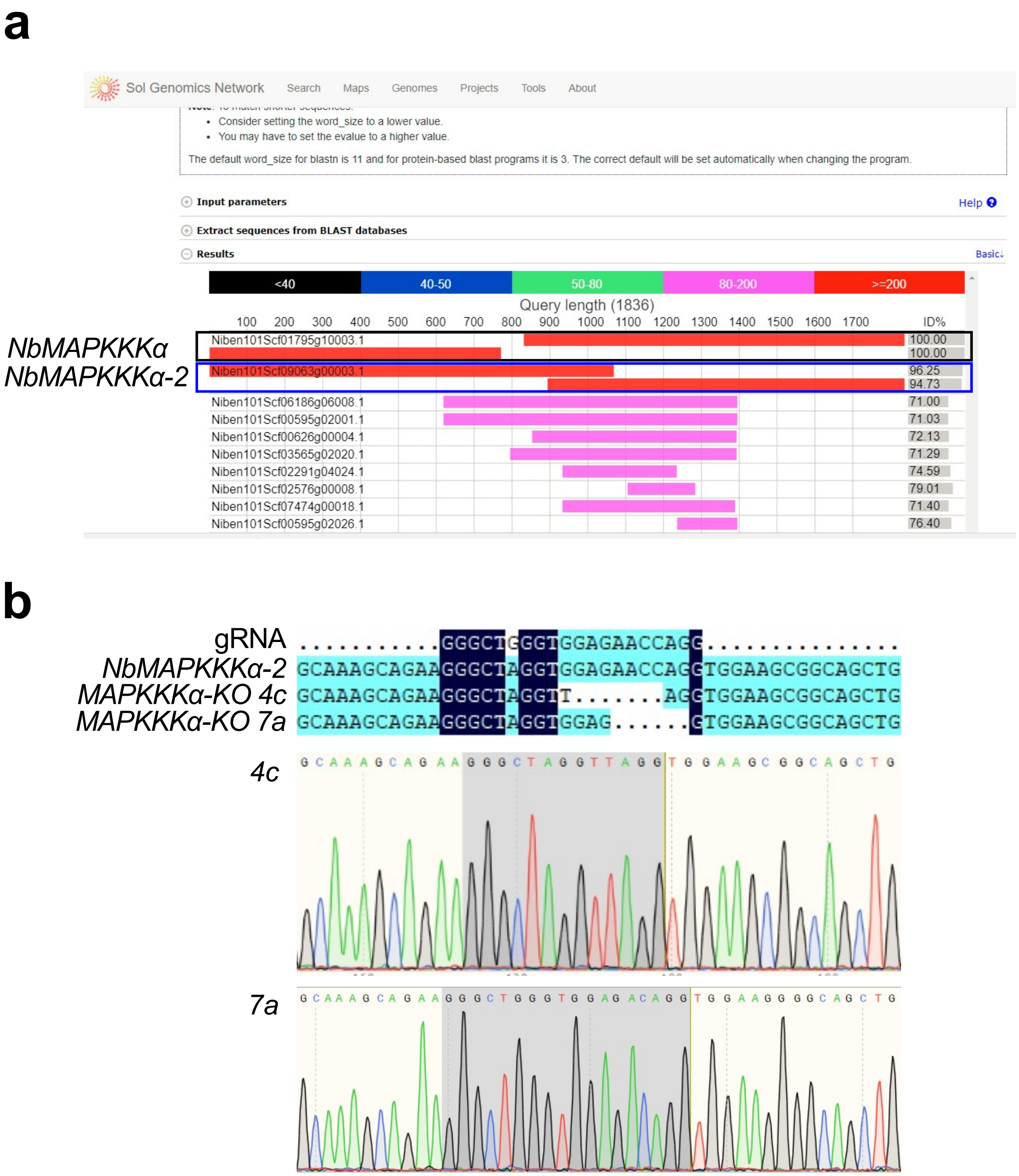

Supplementary Figure 1. MAPKKKα showed two homologs in *N. benthamiana* sequence database, related to Fig. 1e.

a. The amino acid sequences of MAPKKKα and its homologs were obtained by blasting the MAPKKKα protein sequence against the *N. benthamiana* sequence database (https://solgenomics.net/tools/blast/).

b. Sequencing of MAPKKKα homolog (MAPKKKα-2) in two *NbMAPKKKα*-KO lines.

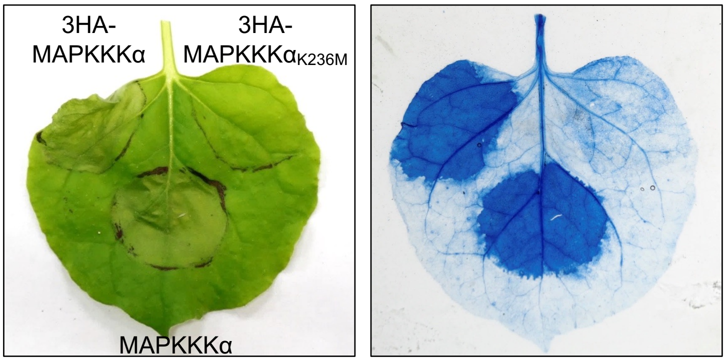

Supplementary Figure 2. The kinase-inactive MAPKKKα$_{K236M}$ does not induce cell death in *N. benthamiana* leaves, related to Fig. 1g.
The representative photographs were taken at 3 dpi.

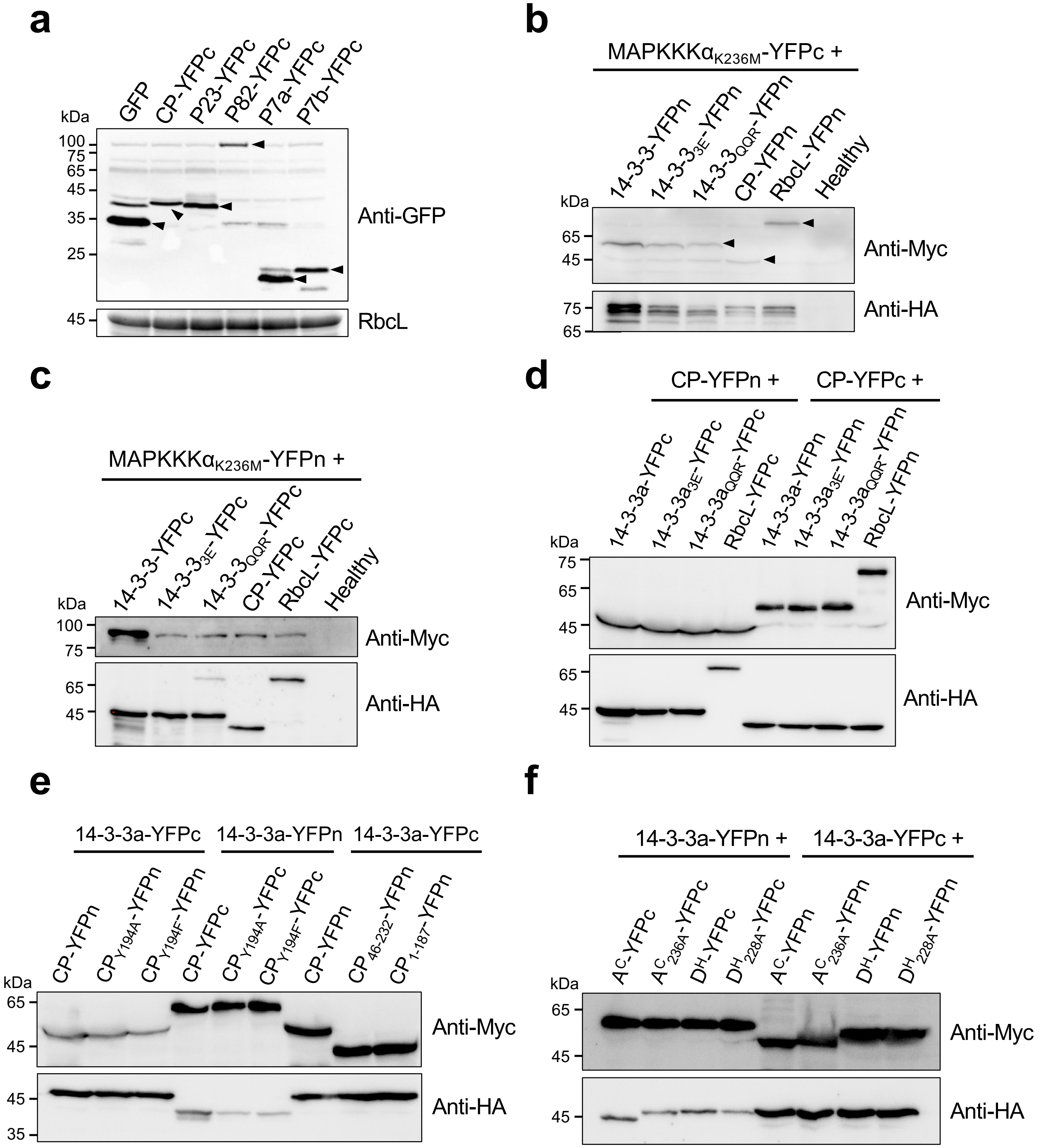

Supplementary Figure 3. Western blot analysis of the target protein expression in the infiltrated leaves.

a. Analysis of protein expression in the leaves shown in Fig. 3a by Western blot with an anti-GFP antibody. RbcL served as the loading control.

b-f. Western blot analysis of target protein expression in the BiFC assays shown in Supplementary Fig. 4a and Figs. 3g, 4a, 6b, 6d, 9a and 9b with anti-Myc or anti-HA antibodies. All the experiments were repeated three times with similar results.

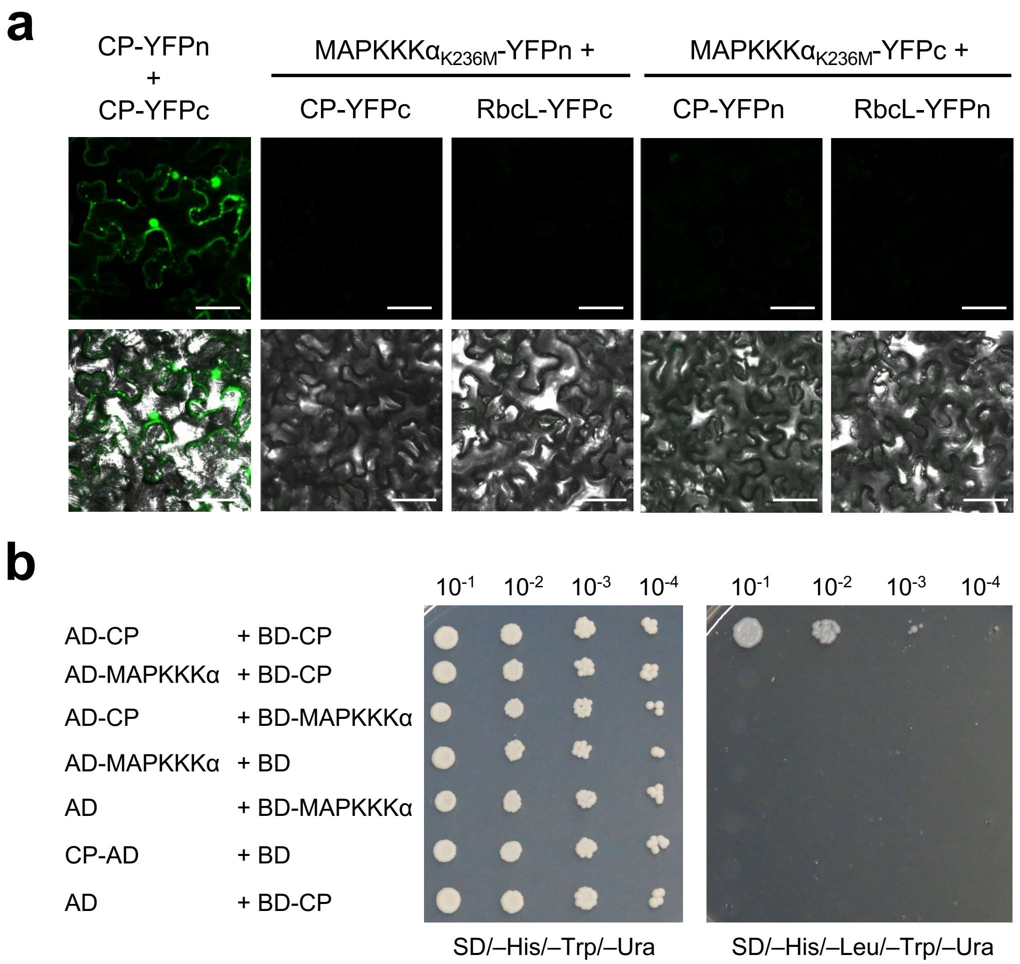

Supplementary Figure 4. CP does not interact with MAPKKKα, related to Fig. 3.

a. BiFC assay to test the interaction between CP and MAPKKKα. Indicated combination of different proteins were transiently expressed in *N. benthamiana* leaves. Confocal analysis was performed at 3 dpi. CP-YFPn and CP-YFPc served as the positive control. RbcL protein served as negative controls. Scale bars = 50 µm. The experiment was repeated three times with similar results.

b. Yeast two-hybrid (Y2H) assay to evaluate the interactions between CP and MAPKKKα proteins. Yeast cells transformed with the indicated plasmids were spotted onto yeast SD dropout media (SD/–His/–Trp/–Ura or SD/Gal/Raf/–His/–Leu/–Trp/–Ura) plates, in a series of 10-fold dilutions. The self-interaction of CP proteins was confirmed and served as positive control, whereas the Y2H combinations containing either empty AD or BD constructs served as negative controls.

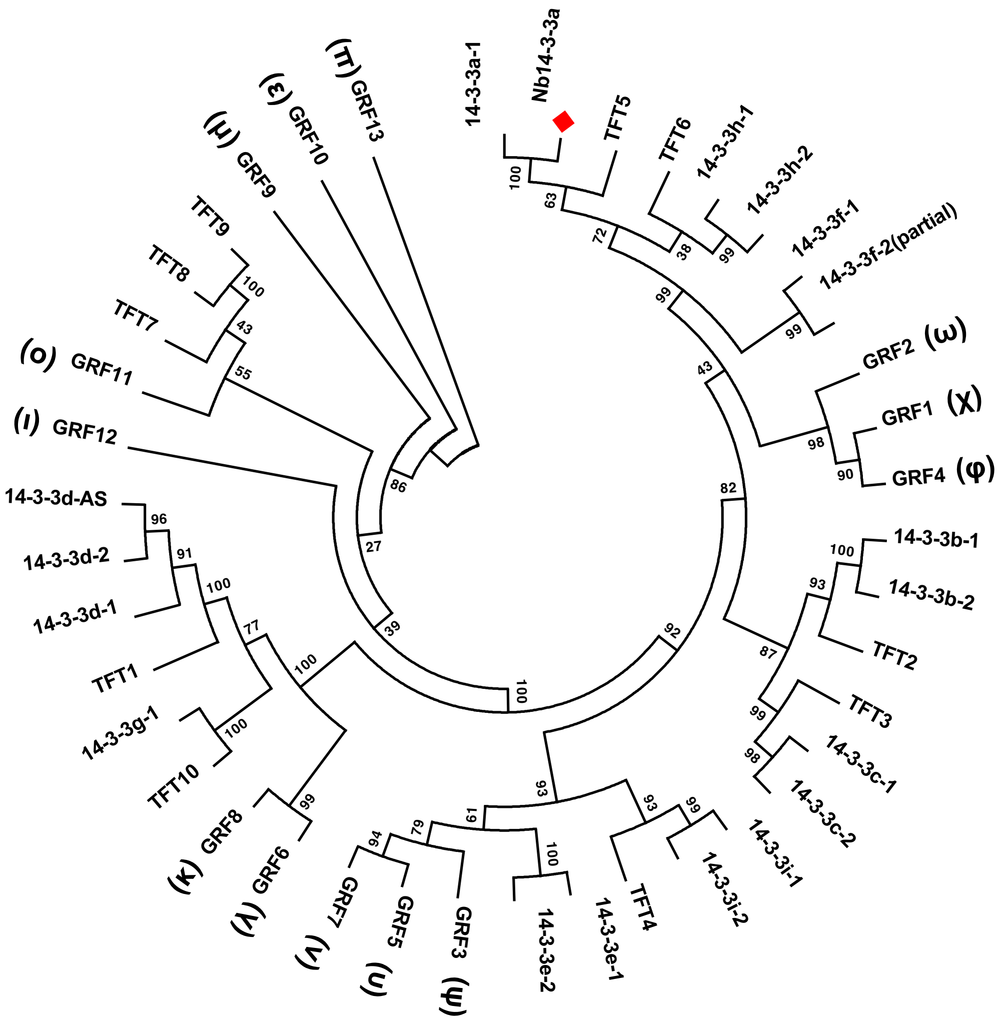

Supplementary Figure 5. Phylogenetic tree of 14-3-3 family from multiple plant species, related to Fig. 3.

The amino acid sequences of 14-3-3 isoforms from tobacco, tomato and *Arabidopsis* were reported previously[42]. 14-3-3a-i indicate 14-3-3 isoforms in tobacco, TFT1-10 indicate 14-3-3 isoforms in tomato, and GRF1-13 indicate 14-3-3 isoforms in *Arabidopsis*. The sequences were aligned and the phylogenetic tree was generated by DNAMAN (version 8.0).

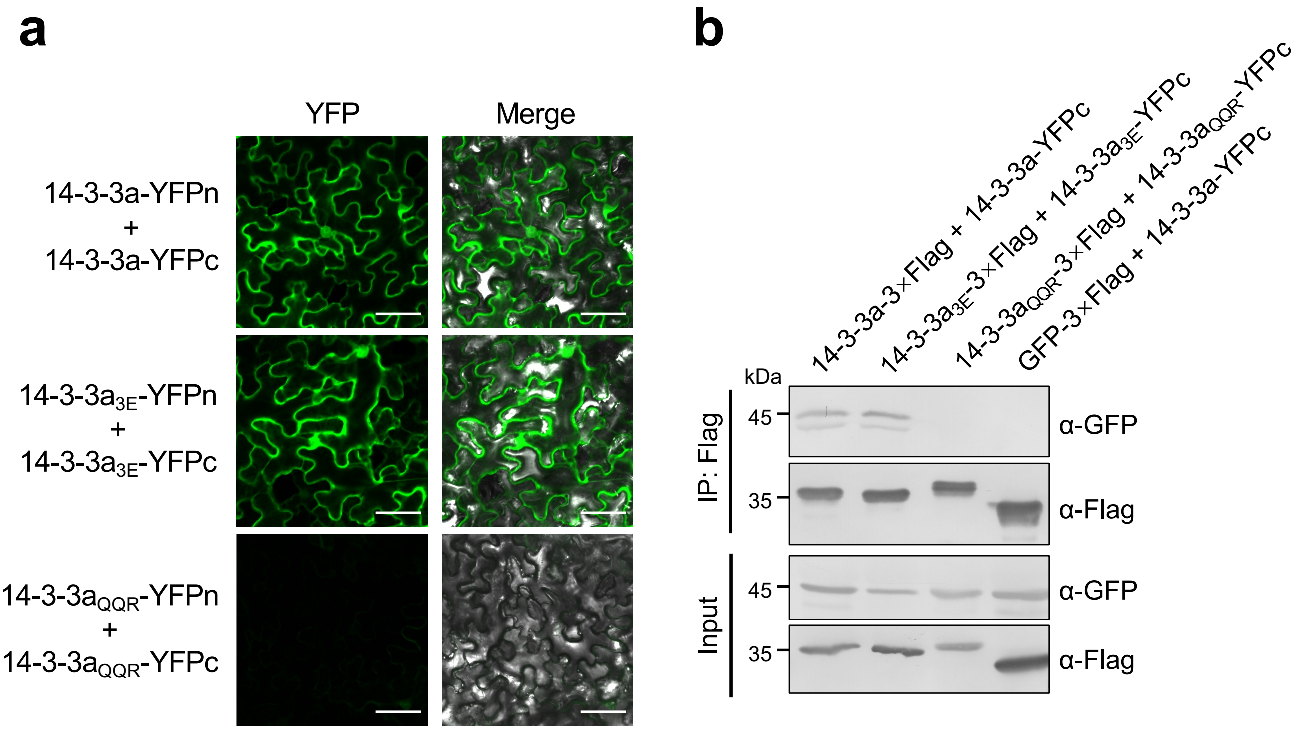

Supplementary Figure 6. The amino acid sequence 12-LAE-14 within the 14-3-3a is critical for its dimerization, related to Fig. 3.

a. BiFC assay to test the self-interaction of wild-type or mutant 14-3-3a. Combinations of different proteins were transiently co-expressed in *N. benthamiana* leaves. Confocal analysis was performed at 3 dpi. Scale bars = 50 µm.

b. Co-IP analysis of the self-interaction of wild-type or mutant 14-3-3a. *N. benthamiana* leaves transiently expressing combinations of different proteins were harvested at 3 dpi. Total proteins were immunoprecipitated with anti-Flag beads and analyzed by Western blot with an anti-GFP or anti-Flag antibody. All the experiments were repeated three times with similar results.

.

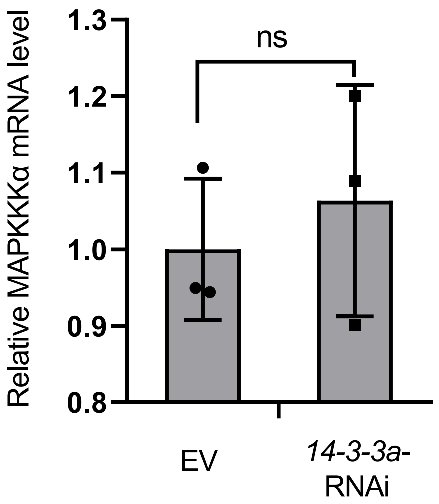

Supplementary Figure 7. Silencing 14-3-3a has little effects on MAPKKKα transcripts, related to Fig. 4.
Leaves infiltrated with *Agrobacterium* containing hairpin 14-3-3a or the control empty vector (EV) were sampled at 3 dpi. Values represent ± SD of the mean from three biological replicates. *EF1α* was used as the internal reference gene to normalize the relative expression.

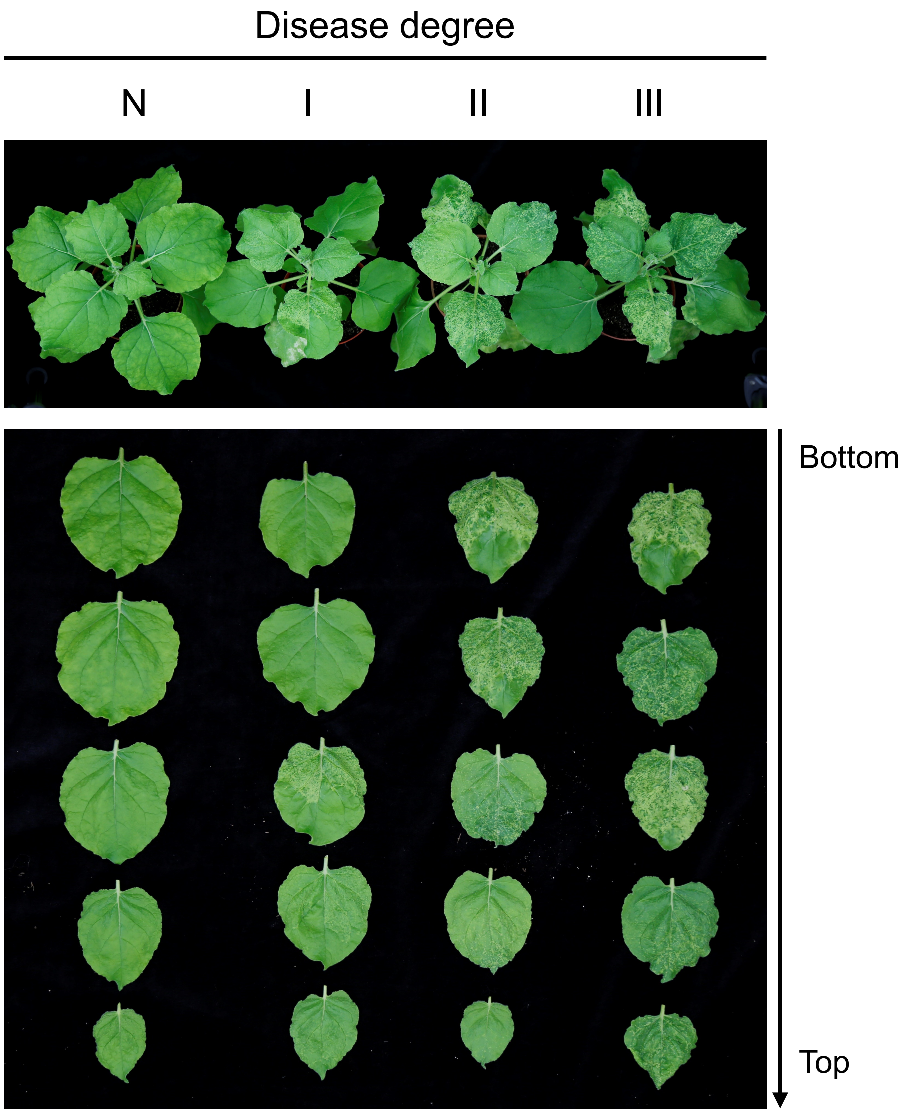

Supplementary Figure 8. Photographs of BBSV-infected *N. benthamiana* plants with different degrees of symptom severity, related to Fig. 5d. N indicates leaves without observable symptom.

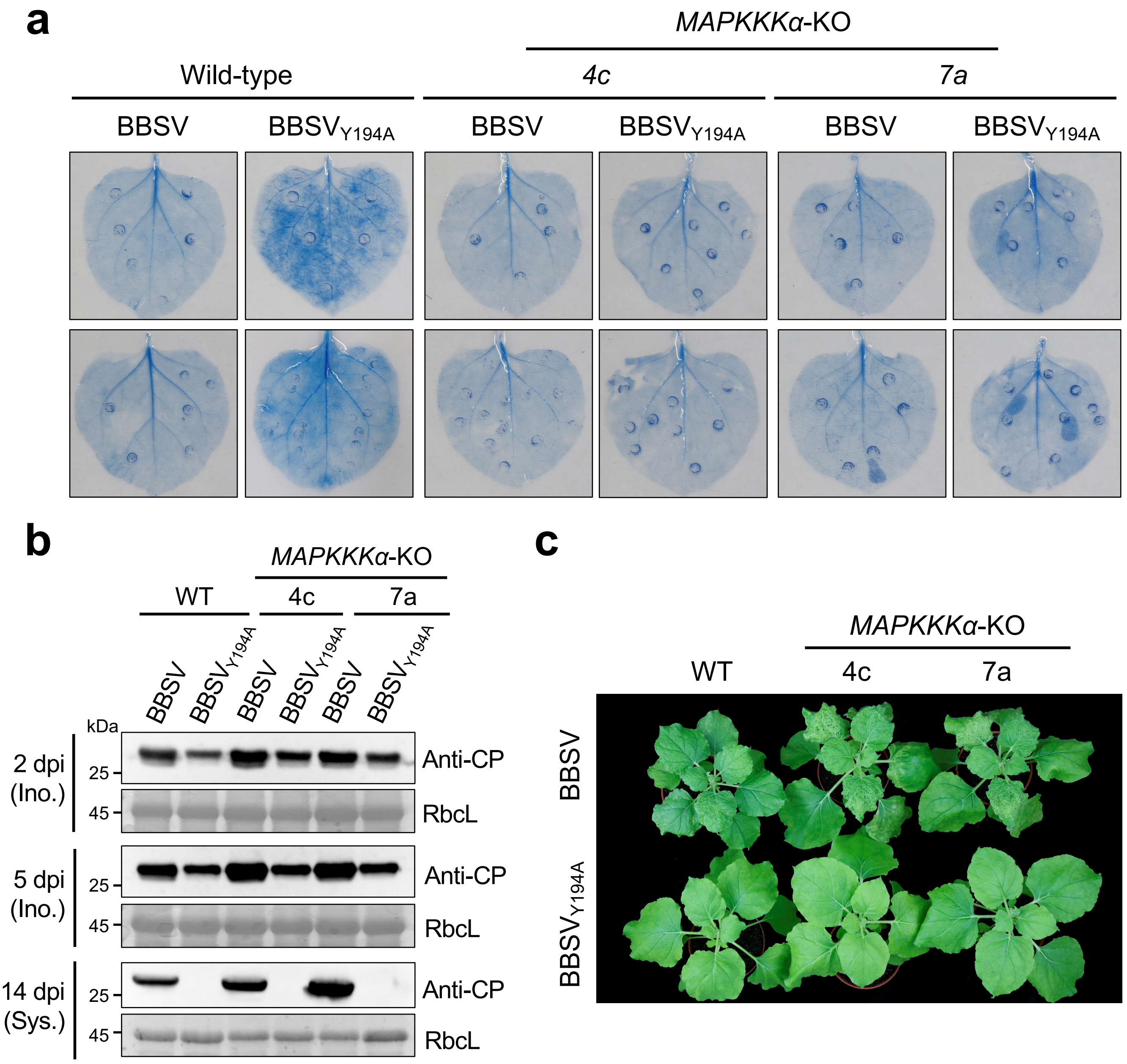

Supplementary Figure 9. Analysis of the infection of WT BBSV or BBSV$_{Y194A}$ in WT or *MAPKKKα*-KO *N. benthamiana* plants, related to Fig. 7.

a. Trypan blue staining of WT or *MAPKKKα*-KO leaves inoculated with BBSV or BBSV$_{Y194A}$ at 2 dpi. Representative results of three independent experiments are shown.

b. Western blot analysis of CP accumulation in the local or systemic leaves infected with BBSV or BBSV$_{Y194A}$. The experiment was repeated three times with similar results.

c. Systemic symptoms of *N. benthamiana* plants infected with BBSV or BBSV$_{Y194A}$. Representative results of two independent experiments are shown.

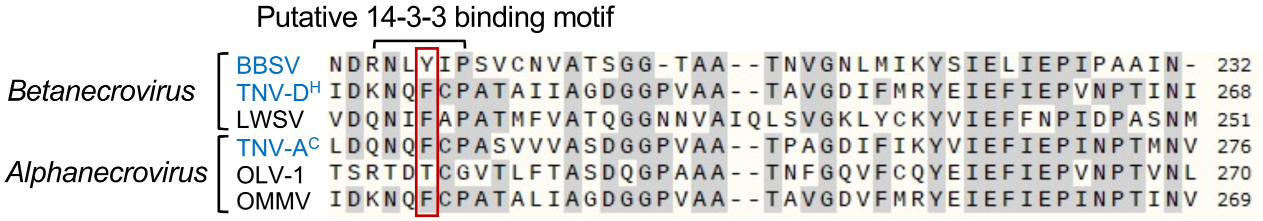

Supplementary Figure 10. Amino acid sequence alignment of C-terminal regions of viral CP proteins from two different genera, related to Fig. 9.

The sequences were obtained from NCBI protein database and aligned using UniPort (https://www.uniprot.org/align/). The red box region highlights the amino acids which are supposed to be critical for 14-3-3a binding. Amino acids under black lines indicate putative 14-3-3 binding motifs. The aligned viruses are: TNV-D^{H}, Hungarian isolate; LWSV, leek white stripe virus; TNV-A^{C}, tobacco necrosis virus-A Chinese isolate; TCV, Turnip crinkle virus; MNSV, melon necrotic spot virus; OLV-1, olive latent virus 1; OMMV, olive mild mosaic virus.

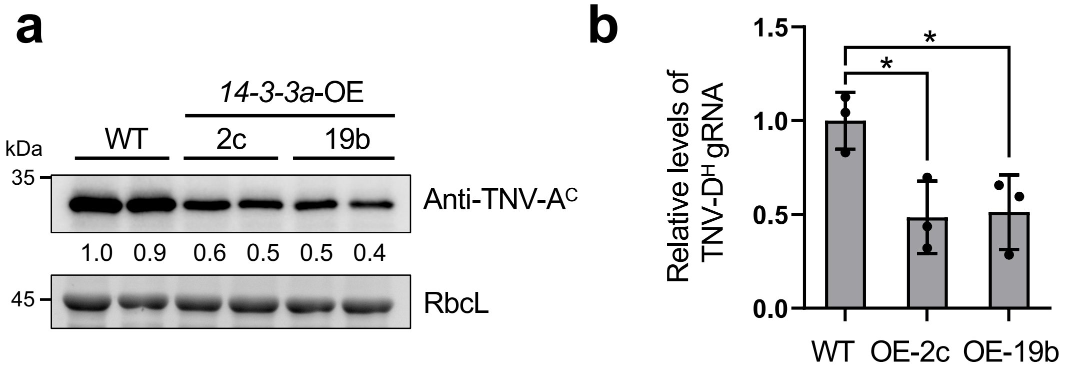

Supplementary Figure 11. Nb14-3-3a showed antiviral defense against two representative viruses from genera *Betanecroviruses* and *Alphanecroviruses*, related to Fig. 9.

a. Western blot analysis of TNV-A^C CP levels using an anti-TNV-A^C antibody. RbcL served as the loading control. The experiment was repeated three times with similar results.

b. RT-qPCR analysis of TNV-D^H genomic RNA levels. Values represent ± SD of the mean from three biological replicates. An asterisk indicates the significant difference based on one-way ANOVA analysis with Dunnett's multiple comparison test (**P* = 0.0238 and 0.0299 respectively).

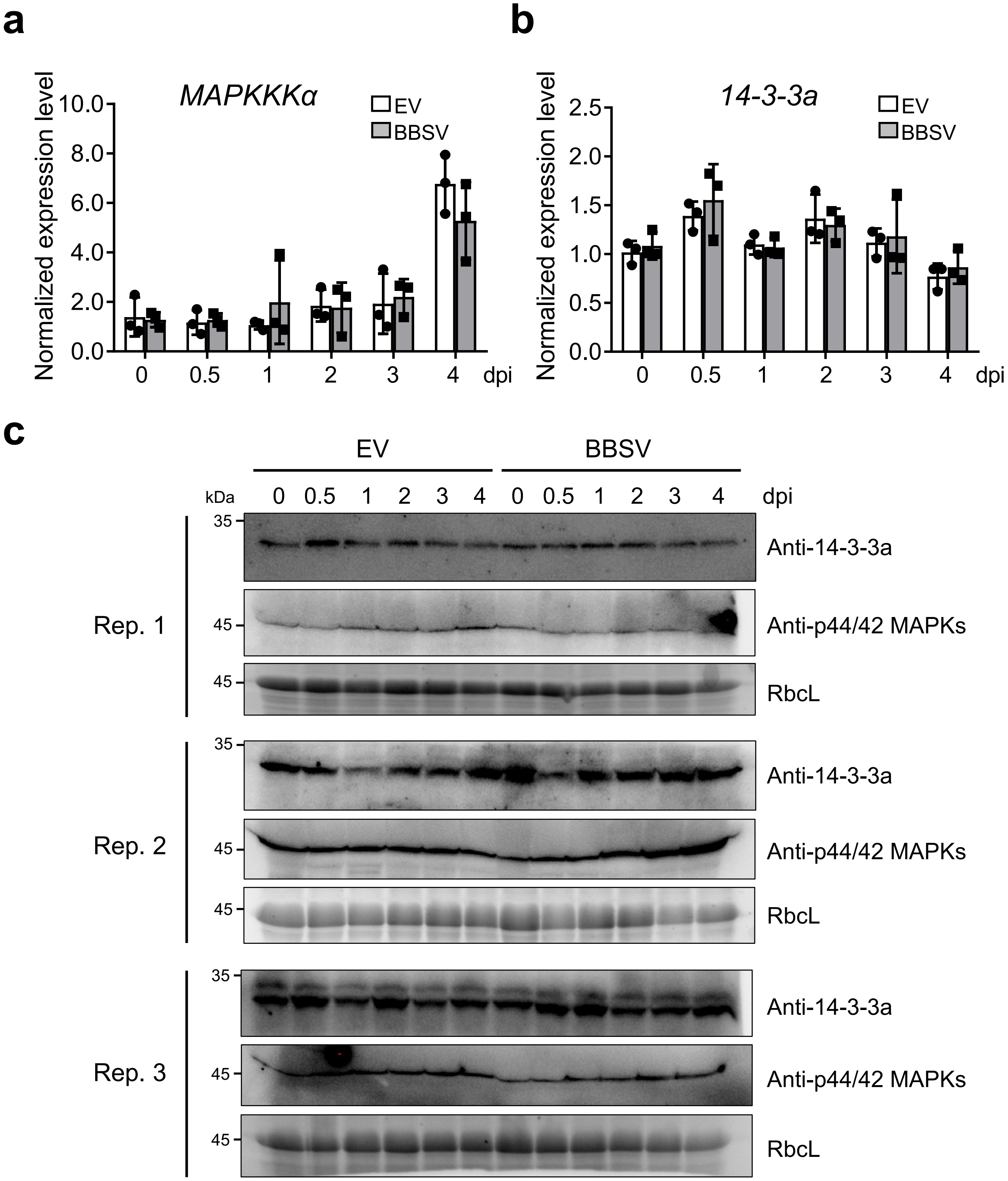

Supplementary Figure 12. BBSV infection does not affect the expression levels of Nb14-3-3a and MAPKKKα, related to Figs. 1 and 5.

a-b. RT-qPCR analysis of MAPKKKα (a) and 14-3-3a (b) mRNA levels in EV- or BBSV-inoculated *N. benthamiana* at indicated time points. Values represent ± SD of the mean from three biological replicates. *EF1α* was used as the internal reference gene to normalize the relative expression.

c. Western blot analysis of 14-3-3a and MAPKs (SIPK and WIPK) protein levels at different timepoints using anti-14-3-3a and anti-p44/42 MAPKs antibodies. RbcL served as the loading control. The experiment was repeated three times with similar results.
