# Supplementary material for: Coat proteins of necroviruses target 14-3-3a to subvert MAPKKKα-mediated antiviral immunity in plants
Source: Nat Commun. 2022 Feb 7;13:716. doi: 10.1038/s41467-022-28395-5 (PMC8821596; doi:10.1038/s41467-022-28395-5)
Supplement: Supplementary file 2 — Description of Additional Supplementary Files [file 41467_2022_28395_MOESM2_ESM.pdf]

Description of Additional Supplementary Files

File name: Supplementary Data 1

Description: Up-regulated genes at 0.5, 1, 2 dpi in WT N*. benthamiana* plants during BBSV infection.

File name: Supplementary Data 2

Description: 370 genes that were up-regulated in WT N. *benthamiana* plants after BBSV infection but were downregulated in the comparisons between KO-BBSV and WT-BBSV.

File name: Supplementary Data 3

Description: Proteins identified by LC-MS/MS after immunoprecipitation of CP-3×Flag proteins from BBSV-infected N. *benthamiana*.

File name: Supplementary Data 4

Description: Transcription analysis of MAPKKKα at 0.5, 1, 2 dpi in WT N. *benthamiana* plants.

File name: Supplementary Data 5

Description: Transcription analysis of 14-3-3a at 0.5, 1, 2 dpi in WT N. *benthamiana* plants.

File name: Supplementary Data 6

Description: Primers used in this study.
