# Supplementary material for: Coat proteins of necroviruses target 14-3-3a to subvert MAPKKKα-mediated antiviral immunity in plants
Source: Nat Commun. 2022 Feb 7;13:716. doi: 10.1038/s41467-022-28395-5 (PMC8821596; doi:10.1038/s41467-022-28395-5)
Supplement: Supplementary file 9 — Reporting Summary [file 41467_2022_28395_MOESM9_ESM.pdf]

nature portfolio

Corresponding author(s): Yongliang Zhang

Last updated by author(s): Dec 30, 2021

Reporting Summary

Nature Portfolio wishes to improve the reproducibility of the work that we publish. This form provides structure for consistency and transparency in reporting. For further information on Nature Portfolio policies, see our Editorial Policies and the Editorial Policy Checklist.

Statistics

For all statistical analyses, confirm that the following items are present in the figure legend, table legend, main text, or Methods section.

n/a	Confirmed	
☐	☒	The exact sample size (*n*) for each experimental group/condition, given as a discrete number and unit of measurement
☐	☒	A statement on whether measurements were taken from distinct samples or whether the same sample was measured repeatedly
☐	☒	The statistical test(s) used AND whether they are one- or two-sided<br>*Only common tests should be described solely by name; describe more complex techniques in the Methods section.*
☒	☐	A description of all covariates tested
☐	☒	A description of any assumptions or corrections, such as tests of normality and adjustment for multiple comparisons
☐	☒	A full description of the statistical parameters including central tendency (e.g. means) or other basic estimates (e.g. regression coefficient) AND variation (e.g. standard deviation) or associated estimates of uncertainty (e.g. confidence intervals)
☐	☒	For null hypothesis testing, the test statistic (e.g. *F*, *t*, *r*) with confidence intervals, effect sizes, degrees of freedom and *P* value noted<br>*Give P values as exact values whenever suitable.*
☒	☐	For Bayesian analysis, information on the choice of priors and Markov chain Monte Carlo settings
☐	☒	For hierarchical and complex designs, identification of the appropriate level for tests and full reporting of outcomes
☒	☐	Estimates of effect sizes (e.g. Cohen's *d*, Pearson's *r*), indicating how they were calculated

Our web collection on statistics for biologists contains articles on many of the points above.

Software and code

Policy information about availability of computer code

Data collection	Sol Genomics Network (https://solgenomics.net/), Nicotiana benthamiana transcriptome database (http://benthgenome.qut.edu.au/), National Center for Biotechnology Information (NCBI), Illumina NovaSeq 6000, Q-Exactive mass spectrometer (Thermo Fisher Scientific), Zeiss LSM880, CFX Manger (Bio-Rad)
Data analysis	DESeq2 R package (1.20.0), Hisat2 (v2.0.5), StringTie (v1.3.3b), CRISPR-P 2.0 design tool (http://crispr.hzau.edu.cn/CRISPR2/), GraphPad Prism 8, Office 2016, ImageJ, Mascot Server (version 2.5.1, Matrix Science), DNAMAN (version 8.0), UniPort (https://www.uniprot.org/align/)

For manuscripts utilizing custom algorithms or software that are central to the research but not yet described in published literature, software must be made available to editors and reviewers. We strongly encourage code deposition in a community repository (e.g. GitHub). See the Nature Portfolio guidelines for submitting code & software for further information.

Data

Policy information about availability of data

All manuscripts must include a data availability statement. This statement should provide the following information, where applicable:

- Accession codes, unique identifiers, or web links for publicly available datasets
- A description of any restrictions on data availability
- For clinical datasets or third party data, please ensure that the statement adheres to our policy

All data generated or analysed during this study are included in this published article (and its supplementary information files)

Field-specific reporting

Please select the one below that is the best fit for your research. If you are not sure, read the appropriate sections before making your selection.

☒ Life sciences ☐ Behavioural & social sciences ☐ Ecological, evolutionary & environmental sciences

For a reference copy of the document with all sections, see nature.com/documents/nr-reporting-summary-flat.pdf

Life sciences study design

All studies must disclose on these points even when the disclosure is negative.

Sample size	For local viral infection assays, >3 plants per group were used.<br>For systemic viral infection assays, >6 plants per group were used.<br>For gene expression assays, >5 plants per group were used.<br>For RNA-Seq analysis, 3 samples per group were used.
Data exclusions	No data were excluded.
Replication	The key experiments were repeated at least three times with similar results, as indicated in the figure legends, The original data for these replicates are provided in the Source data file.
Randomization	Due to the nature of the experimental setup, randomization was not practical.
Blinding	Due to the nature of the experimental setup, blinding was not practical.

Reporting for specific materials, systems and methods

We require information from authors about some types of materials, experimental systems and methods used in many studies. Here, indicate whether each material, system or method listed is relevant to your study. If you are not sure if a list item applies to your research, read the appropriate section before selecting a response.

Materials & experimental systems

n/a	Involved in the study	
☐	☒	Antibodies
☒	☐	Eukaryotic cell lines
☒	☐	Palaeontology and archaeology
☒	☐	Animals and other organisms
☒	☐	Human research participants
☒	☐	Clinical data
☒	☐	Dual use research of concern

Methods

n/a	Involved in the study	
☒	☐	ChIP-seq
☒	☐	Flow cytometry
☒	☐	MRI-based neuroimaging

Antibodies

Antibodies used	1. Anti-BBSV CP antibody, Rabbit pAb, used in Western blot, produced by Beijing Protein Innovation Co., Ltd.<br>2. Anti-14-3-3a antibody, Rabbit pAb, used in Western blot, produced by Beijing Protein Innovation Co., Ltd.<br>3. Anti-Myc-tag antibody, Mouse mAb, used in Western blot, MBL, Code: M047-3.<br>4. Anti-HA-tag antibody, Mouse mAb, used in Western blot, MBL, Code: M180-3.<br>5. Anti-GFP antibody, Rabbit pAb, used in Western blot, MBL, Code: 598.<br>6. Anti-His Tag antibody, Mouse mAb, used in Western blot, EASYBIO, Cat#: BE7001.<br>7. Anti-FLAG M2 antibody, Mouse mAb, used in Western blot, Sigma, Cat#: F1804.<br>8. Anti-GST antibody, Mouse mAb, used in Western blot, Genscript, Cat#: A00866.<br>9. p44/42 MAPK (Erk1/2) (137F5) Rabbit mAb, used in Western blot, Cell Signaling, Cat#: 4695.<br>10. Phospho-p44/42 MAPK (Erk1/2) (Thr202/Tyr204) (D13.14.4E) XP Rabbit mAb, used in Western blot, Cell Signaling, Cat#: 4370.<br>11. Goat Anti-Mouse IgG, used in Western blot, Abbkine, Cat#: A21110.<br>12. Goat Anti-Rabbit IgG H&L (HRP), used in Western blot, EASYBIO, Cat#: BE0101.
Validation	1. Anti-Myc-tag antibody, Mouse mAb, manual can be found at 'http://www.mbl-chinawide.cn/search-details2?id=21058&table=RuoAntibody'.<br>2. Anti-HA-tag antibody, Mouse mAb, manual can be found at 'http://www.mbl-chinawide.cn/search-details2?id=21067&table=RuoAntibody'.<br>3. Anti-GFP antibody, Rabbit pAb, manual can be found at 'http://www.mbl-chinawide.cn/search-details2?id=21052&table=RuoAntibody'.<br>4. Anti-His Tag antibody, Mouse mAb, manual can be found at 'http://www.bioeasytech.com/home/product/article/id/565/sear/HIS.html'.

5. Anti-FLAG M2 antibody, Mouse mAb, manual can be found at 'https://www.sigmaaldrich.cn/CN/en/search/flag-f1804?focus=products&page=1&perPage=30&sort=relevance&term=FLAG%20F1804&type=product'.
6. Anti-GST antibody, Mouse mAb, manual can be found at 'https://www.genscript.com.cn/antibody/A00866-THE_GST_Antibody_HRP_mAb_Mouse.html?page_no=1&position_no=1&sensors=search'.
7. p44/42 MAPK (Erk1/2) (137F5) Rabbit mAb, manual can be found at 'https://www.cellsignal.cn/products/primary-antibodies/p44-42-mapk-erk1-2-137f5-rabbit-mab/4695?site-search-type=Products&N=4294956287&Ntt=4695&fromPage=plp'.
8. Phospho-p44/42 MAPK (Erk1/2) (Thr202/Tyr204) (D13.14.4E) XP Rabbit mAb, manual can be found at 'https://www.cellsignal.cn/products/primary-antibodies/phospho-p44-42-mapk-erk1-2-thr202-tyr204-d13-14-4e-xp-rabbit-mab/4370?site-search-type=Products&N=4294956287&Ntt=4370&fromPage=plp'.
